# Matriline effects on metamorphic traits in a natural system in the European common frog (*Rana temporaria*)

**DOI:** 10.1002/ece3.4811

**Published:** 2019-02-21

**Authors:** Carolin Dittrich, Juliane Huster, Mark‐Oliver Rödel, Heike Feldhaar

**Affiliations:** ^1^ Museum für Naturkunde Leibniz Institute for Evolution and Biodiversity Science Berlin Germany; ^2^ Animal Ecology I, Bayreuth Center for Ecology and Environmental Research (BayCEER) University of Bayreuth Bayreuth Germany; ^3^ Berlin‐Brandenburg Institute of Advanced Biodiversity Research (BBIB) Berlin Germany

**Keywords:** amphibians, genetic effects, microsatellites, multiple paternity, natural selection

## Abstract

Successful reproduction is an important determinant of the fitness of an individual and of the dynamics of populations. Offspring of the European common frog (*Rana temporaria*) exhibit a high degree of variability in metamorphic traits. However, environmental factors alone cannot explain this phenotypic variability, and the influence of genetic factors remains to be determined. Here, we tested whether the maternal genotype influences developmental time, body size, and body condition of offspring in a forest pond in Germany. We collected fertilized eggs from all 57 clutches deposited in the pond. We used multilocus genotypes based on seven microsatellite loci to assign metamorphosed offspring to mothers and to determine the number of fathers for a single matriline. We tested the influence of genetic effects in the same environment by comparing variability of metamorphic traits within and between full‐sib offspring grouped to matrilines and tested whether multiple paternity increases the variability of metamorphic traits in a single matriline. The variability in size and body condition was higher within matrilines than between them, which indicates that these traits are more strongly influenced by environmental effects, which are counteracting underlying genetic effects. The developmental time varied considerably between matrilines and variability increased with the effective number of fathers, suggesting an additive genetic effect of multiple paternity. Our results show that metamorphic traits are shaped by environmental as well as genetic effects.

## 1 INTRODUCTION

Natural selection shapes life‐history traits of individuals, which optimizes fitness in a given environment. Under optimality theory, the timing of specific life‐history events, such as reproduction or metamorphosis, will evolve to an optimum due to natural selection and local adaptation (Parker & Smith, 1990). Metamorphosis, as an important life‐history event, has received a lot of attention, with different models developed to explain the best possible transition and niche shift under different environmental conditions in complex life cycles (Rowe & Ludwig, 1991; Rudolf & Rödel, 2007; Wilbur & Collins, 1973).

In natural amphibian populations, the metamorphic traits of individuals (e.g., size, body condition, age at metamorphosis, and developmental time) can differ profoundly within and between populations (Grözinger, Thein, Feldhaar, & Rödel, 2014; Loman, 2004). Factors known to influence these traits under laboratory conditions are temperature, food availability, intra‐ and interspecific competition, presence of predators, and seasonal time constraints (Drakulić et al., 2016; Laugen, Laurila, Räsänen, & Merilä, 2003; Laurila & Kujasalo, 1999; Merilä, Laurila, Pahkala, Räsänen, & Laugen, 2000; Pakkasmaa & Aikio, 2003; Smith‐Gill & Berven, 1979; Van Buskirk, 2017). Little is known of the interacting effects in natural environments (Loman, 2001, 2004), where environmental variables often seem to counteract the genetic effects. This process, known as countergradient variation, can occur on small geographical scales (Conover & Schultz, 1995; Dittrich, Drakulić, Schellenberg, Thein, & Rödel, 2016; Laugen et al., 2003; Skelly, 2004). Genetic effects have been shown to influence metamorphic traits mainly by high dominance and additive effects, particularly, age at metamorphosis and growth rate (Laugen et al., 2005; Laurila, Karttunen, & Merilä, 2002). In addition, maternal effects like egg size or egg provisioning, could influence metamorphic traits, but were shown to be mostly weak (Laugen et al., 2005) and seem to be highly dependent on the environment. Furthermore, females are able to follow different life‐history strategies concerning the age and/or size of first reproduction and could adjust their strategies throughout their reproductive lifetime. Females may allocate their reproductive investment either into a larger quantity (many but small offspring) or quality (fewer but larger offspring) of progeny (“offspring number‐size trade‐off”; Smith & Fretwell, 1974; Charnov & Ernest, 2006).

The European common frog (*Rana temporaria* Linneaus, 1758) is one of the most widespread amphibians in Central and Northern Europe (Sillero et al., 2014). This generalist species expresses high variability and phenotypic plasticity in metamorphic traits (Grözinger, Feldhaar, Thein, & Rödel, 2018; Laurila, Pakkasmaa, & Merilä, 2001; Ryser, 1996; Ståhlberg, Olsson, & Uller, 2001).

Additionally, multiple paternity was shown to occur in this species, either as a consequence of stray sperm (Laurila & Seppä, 1998) or of “clutch piracy” (Vieites et al., 2004). Multiple paternity could increase genetic variability among offspring and thereby increase viability of offspring (Jennions & Petrie, 2000). In laboratory studies, a sire effect on developmental time and survival was found (Laugen, Laurila, & Merilä, 2002; Merilä, Laurila, Pahkala, et al., 2000). In this study, we investigate the influence of maternal genotypes and putative effects of multiple paternity on post‐metamorphic traits and trait variability within one natural pond. To our knowledge, the assignment of anuran metamorphs to their respective matrilines with molecular techniques is unique and the first study of its kind. All individuals share the same environment and therefore environmental effects, which could influence metamorphic traits. Microsatellite analysis was used to assign full‐ and half‐siblings to a single mother (matriline) and determine the number of fathers. Furthermore, we examined the effect of multiple paternity on the variability of metamorphic traits of the progeny within matrilines.

We tested the following hypotheses:

1. Offspring from different mothers show high variability in metamorphic traits between matrilines within one shared environment, due to maternal and paternal genetic effects.
2. An increased number of sires of one clutch should increase the variability in metamorphic traits within the respective matriline due to additive genetic effects.
3. Some matrilines are more successful in reproduction than others due to faster offspring development, bigger offspring and higher offspring numbers in the same environment, due to different resource provisioning.

## 2 MATERIAL AND METHODS

### 2.1 Site and sampling of clutches

Clutch samples and metamorphs of *R. temporaria* were collected from a pond in the northern Steigerwald (Bavaria, Germany), near the village of Fabrikschleichach (49°54′N, 10°32′E). From the 1970s, 120 small artificial ponds were constructed in this 28 km^2^ area for conservation purposes by the state forestry department. Our study pond has a surface of 12 m^2^ and is located in a 28 km^2^ beech grove and mixed forest which has been monitored for *R. temporaria* breeding sites since 2005 (Grözinger, Wertz, Thein, Feldhaar, & Rödel, 2012). The maximum depth of the surplus water in the middle of the pond is approx. 50 cm throughout the year. Clutches were deposited within the patchy vegetation on the shallow southern part of the pond (Supporting Information Figure S1). The water temperature was measured with a Thermochron iButton^©^ (accuracy ±0.5°C), and average daily values are given in Supporting Information Figure S2. Additionally, data on local precipitation were obtained from a weather station 2.5 km from the study pond (Supporting Information Figure S2). The pond was checked daily for new clutches from 1st of April until 12th of April. Although the first clutches were already found on 1st of April, we believe that these embryos experienced only marginal (if any) developmental advantage, due to an unusual cold period from 1st to 8th of April. In this period, maximum daily temperatures reached 3°C, at which developmental progress ceases (Loman, 2002). The first hatchlings were observed on 20th of April. During the yearly monitoring of clutches from 2005 to 2018, we found a range of 19 to 103 clutches per year for this specific pond. In the close surroundings (500 m radius), 21 ponds are present, six of which are regularly used for spawning by the common frog (in more than six out of 13 years). The first 30 clutches of *R. temporaria* were found on 1st April 2013, the last clutches were deposited on 12th Apri (total *n* = 57). We sampled 10 eggs each from all clutches and kept them in small plastic containers (Ø 6 cm, 7 cm high) for 48–72 hr at 8°C until the embryos reached Gosner stage 17–20 (Gosner, 1960). Afterward, the embryos were stored in 99% ethanol until further use.

### 2.2 Sampling of metamorphs

To intercept all emerging metamorphs, a fence was installed at the beginning of June 2013 encircling the pond entirely. As soon as metamorphs began leaving the pond, the fence was controlled twice daily (from 8th July to 29th August 2013). Up to 50 metamorphs were captured each day and two measurements were taken: (a) body mass, measured with an electronical balance to the nearest 0.05 g (VOLTKRAFT PS 250) and (b) snout‐vent length (SVL), measured on scale paper with millimeter grid to the nearest 0.5 mm. Two DNA samples were taken by gently swabbing the skin with cotton buds. DNA samples were stored in 1.5 ml reaction tubes containing either 300 µl Cell Lysis Solution (CLS; PUREGENE^®^ DNA Purification Kit; Qiagen) or 300 µl 99% EtOH. All metamorphs were released in a wet area outside the fence. If more than 50 metamorphs emerged per day, measurements were taken from 50 randomly chosen individuals, and all other metamorphs were only counted and released immediately.

### 2.3 Microsatellite analyses

DNA was isolated from four eggs per clutch (*n* = 232 in total) and from 1,176 metamorphs (a maximum of 30 per sampling day) using the PUREGENE^®^ DNA Purification Kit (Qiagen) and stored at −20°C until further use. Individuals were genotyped using microsatellite markers. The microsatellite DNA was amplified via polymerase chain reaction (PCR; details in Supporting Information Table S1) in a total reaction volume of 12.5 µl. We used seven specific primers pairs (BFG046, BFG090, BFG099, BFG203, BFG237, BFG242, BFG250; Matsuba & Merilä, 2009), which were labeled with a fluorescent dye (details in Supporting Information Table S2).

PCR products were analyzed via polyacrylamide gel electrophoresis with a LI‐COR 4300 DNA Analyser (LI‐COR Biosciences). Alleles were scored with saga™ generation 2 automated microsatellite software (LI‐COR Biosciences) and revised manually.

micro‐checker version 2.2.3 (Van Oosterhout, Hutchinson, Wills, & Shipley, 2004) was used to test for null alleles, scoring errors, and large allele dropout. Genotypes with at least five out of seven loci scored (*n* metamorphs = 706) were used for the detection of scoring errors and overall homozygote excess.

For sibship analysis, we used the software colony version 2.0.6.3 (Wang, 2004). The software is based on full‐pedigree likelihood methods to infer sibship among individuals by using multilocus genotype data (Jones & Wang, 2010). Each female is considered to spawn only one clutch per season (Savage, 1961), and we used the genotypes of the clutch samples as additional input to improve sibship assignment as larvae from one clutch represent maternal sibs. The length of the run was set to medium, inbreeding was excluded, and the mating system was set to polygamy for females, because a high proportion of multiple paternity has been shown for *R. temporaria *(Laurila & Seppä, 1998). Offspring sired by the same father but different mothers (half‐sibs) could be genetically more similar than siblings from another matriline; therefore, the mating system for males was set to monogamy to increase differences between matrilines. The allele dropout rate was set to 0.01%, except for the loci BFG046 and BFG242 where a former run of COLONY estimated dropout rates around 0.05%. Allelic dropout occurs when the PCR fails to amplify one of the homologues genes at a locus and therefore could lead to false homozygotes, which could influence the grouping of an individual into a sibship (Wang, 2004). The marker error rate was set to 0.01% for all loci, because these types of errors (false alleles, mutations or contaminant DNA) are less frequent (Wang, 2004). The software arranged the samples of clutch and metamorphs to clusters with a probability of sibship ranging between 0 and 1. Clusters with a probability higher than 0.8 were used for further analysis and defined to represent offspring of a matriline. Some clusters were grouped without clutch sample, which could be due to allelic dropouts that may occur due to the low DNA concentrations we used (Gagneux, Boesch, & Woodruff, 1997). To compare variance in phenotypic traits of offspring within and between matrilines, we only used clusters comprising at least six full‐sibs for further analysis.

### 2.4 Statistical analyses

#### 2.4.1 All emerging metamorphs

All statistical analyses were performed using r statistical software (R Core Team, 2018). To investigate a potential relationship between the SVL and mass of all emerging metamorphs, a Pearson correlation and regression analysis was performed. Because the main spawning time comprised only a few days, all embryos started their development at approximately the same time. Therefore, developmental time was calculated and defined as the time from the beginning of development (median date of spawning activity 10 April 2013) until the end of metamorphosis (day the respective metamorph was collected at the fence). We calculated the body condition index (BCI; scaled mass index after Peig & Green, 2009) of metamorphs. The exponent to calculate the BCI (3.08) was taken from Drakulić et al. (2016), as they studied the same *R. temporaria* population. The measure of body condition gives insights on how well metamorphs are provided with resources to increase the probability of future survival (Scott, Casey, Donovan, & Lynch, 2007). We tested the relationship of SVL and BCI with developmental time using generalized additive models (GAM), because assumptions for linear regression analyses were not met. The models were fitted with restricted maximum likelihood method, and cubic regression splines were used for the explanatory variables SVL and BCI. The GAMs were calculated with the R package mgcv (version 1.8‐24; Wood, 2011). All graphs were drawn with R package ggplot2 (version 3.0.0; Wickham, 2009), and the “jitter” function was used to avoid overplotting.

#### 2.4.2 Multiple paternity and differences in metamorphic traits

To investigate the rate of multiple paternity, we used the mating frequency defined as number of fathers per matriline. To examine the relative proportion of offspring sired by a male, the effective mating frequency (*m*_e_) was calculated (Starr, 1984).

Multiple paternity increases the genetic variability among the offspring of a matriline (Jennions & Petrie, 2000). The influence of multiple paternity on variability in metamorphic traits of the offspring was investigated by comparing two datasets. One dataset (“main father”) contained metamorphs of the main father only (full‐sibs), which we defined as the father who was represented in the clutch sample or in clusters without clutch samples, the father with the highest number of offspring. The second dataset (“all fathers”) contained all metamorphs from all fathers of a matriline (full and half‐sibs).

We used a paired *t* test to investigate whether multiple paternity changes the mean SVL, mean BCI or mean developmental time within matrilines. The coefficient of variation (CV) was used as a measurement of variability of these traits within a matriline. The CV of SVL, BCI, and developmental time for metamorphs from main father and all fathers were calculated for each matriline and compared with a paired *t* test. Single‐mated matrilines were excluded from this analysis. To correct for the different number of metamorphs from main and all fathers of the same matriline, which could affect detected changes in mean or CV due to larger sample size in the all father dataset, we randomly subsampled the same number of metamorphs from main and all fathers 10 times.

#### 2.4.3 Differences in metamorphic traits between matrilines for full‐sibs

After assigning metamorphs to matrilines, we tested if SVL, BCI, and developmental time of full‐sibs (with the same broad genotype) differ between matrilines using the Kruskal–Wallis rank sum test. If metamorphic traits showed significant differences, we performed a post hoc analysis using the Dunn test with *p*‐value correction for multiple testing (false discovery rate; Benjamini & Hochberg, 1995) using the R package fsa (version 0.8.20; Ogle, 2017).

#### 2.4.4 Influence of number of offspring and number of fathers on metamorphic traits

Due to resource partitioning (Smith & Fretwell, 1974), we tested if the number of successfully developing progeny could be related to metamorphic traits, for example, that numerous offspring from one matriline is especially small or large in SVL. If applicable, we used a linear model to see which variables have an influence on mean size and mean BCI of metamorphosed offspring from single matrilines, with number of progeny, number of fathers, and mean developmental time as explanatory variables for the whole dataset (full‐ and half‐sibs). If assumptions for linear regression were not met, we used GAMs.

## 3 RESULTS

### 3.1 **Emigration pattern of** *R. temporaria* **metamorphs**

**Figure 1:**
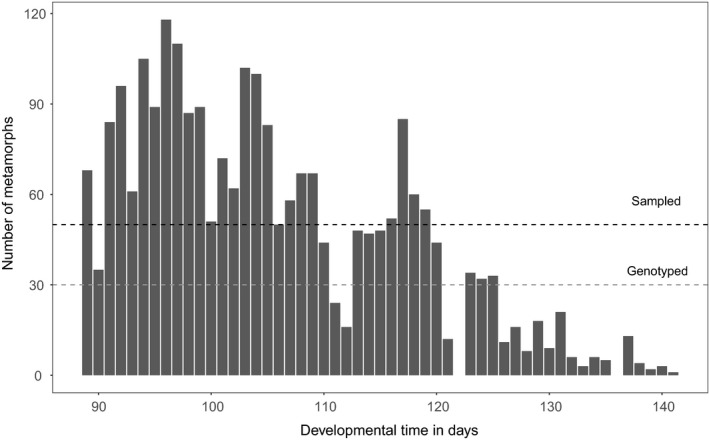
Emigration pattern of *Rana temporaria *metamorphs from one pond in 2013 (8 July–29 August 2013). The black dashed line marks 50 metamorphs (maximum number of sampled metamorphs per day) and the gray dashed line marks 30 metamorphs (maximum number of genotyped metamorphs per day). Missing bars are days without sampling

Overall, 2,414 metamorphs emerged during the whole emigration period (8th July to 29th August 2013). The maximum number of individuals leaving the pond per day was 118 (Figure 1). Given that a clutch contains on average 1,117 eggs (Grözinger et al., 2014) and that we sampled 57 clutches, the survival rate from egg to metamorphosis was 3.8%. Developmental time between metamorphs was highly variable. The majority of the metamorphs (*n* = 1,753; 72%) left within the first 3 weeks of the migration period until day 112 (31 July 2013). The last 28% (*n* = 676) left within the last 4 weeks of the migration period with daily numbers of metamorphs continuously decreasing (Figure 1).

### 3.2 SVL, body condition index, and developmental time of all emigrating metamorphs

**Figure 2:**
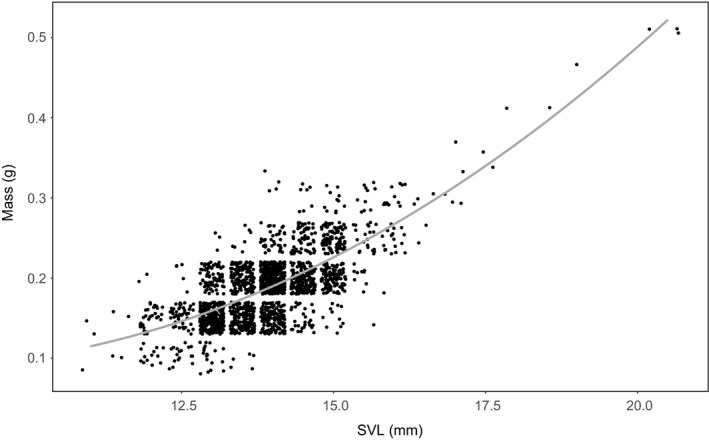
Relationship of SVL (mm) and metamorphic mass (g) of *Rana temporaria* metamorphs (*n* = 1,943) with raw quadratic polynomial function of size to mass (mass = 0.26–0.04 × size + 0.003 × size^2^, gray line). Data points are jittered to avoid overplotting

We measured the SVL, metamorphic mass, and the day of emigration of 1,943 metamorphs (maximum 50 metamorphs per day). The relationship of size and mass was following a nonlinear relation and can be described best by a raw quadratic polynomial function of size on mass (Figure 2; mass = 0.26 − 0.04 × size + 0.003 × size^2^, *df* = 1,940, *p* < 0.001, adjusted *R* ^2^ = 54%). The calculated GAM for metamorphic size as response to developmental time showed a significant, but nonlinear influence of time (SVL increases with time until day 105 and decreases after day 118, Supporting Information Figure S3). Developmental time explained 12.4% of variance in SVL. The GAM for BCI as response to developmental time showed a significant, but nonlinear influence of time (BCI reaches a maximum around day 110, Supporting Information Figure S4) that explained 8.5% of the observed variation in BCI.

### 3.3 Sibship/matriline analyses

To improve the assignment of metamorphs to single matrilines, we genotyped four embryos from each clutch (*n* = 57) and used them as known maternal sibs. From the 2,414 metamorphs that emigrated from the pond, we genotyped 1,176 (maximum 30 per day). In total, 706 metamorphs with five (191 individuals), six (284 individuals), and seven (231 individuals) scored polymorphic microsatellite loci were used for sibship assignment in colony software and the number of alleles ranged from 14 to 25 alleles per locus (Supporting Information Table S3).

The colony software computed 67 clusters based on multilocus genotypes. Ten of these clusters were excluded, because the probability of sibship within the cluster was too low (<0.8). An additional seven clusters were excluded because samples of two or more clutches were clustered together, which could be due to relatedness of spawning females. Of the remaining 50 clusters, 23 were generated without clutch samples. As defined above, a cluster without a clutch sample contained at least six metamorphs from the same father genotype to be designated as a matriline. Thus, 10 of these 23 clusters were excluded. The remaining 40 clusters were defined as matrilines and were used for further analyses (*n* = 439 metamorphs). More details can be found in Appendix S1.

### 3.4 Multiple paternity and differences in metamorphic traits

**Figure 3:**
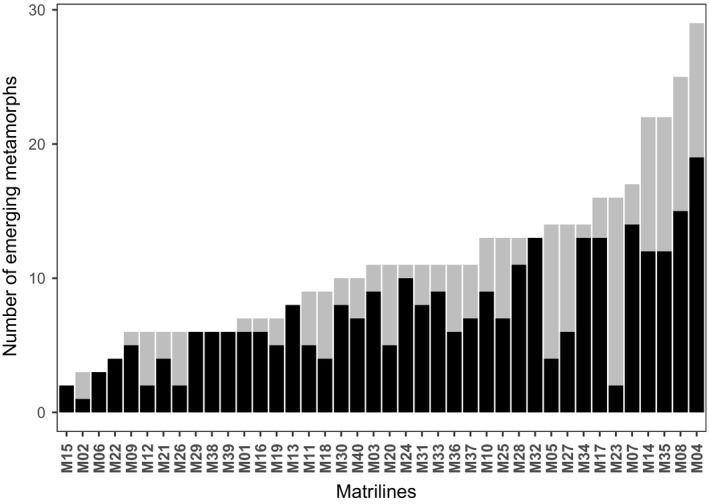
Number of metamorphs per matriline from main father (black, *n* = 294) and all fathers (gray, *n* = 439)

Only eight matrilines exclusively contained full‐sibs, and 32 of all 40 matrilines were fertilized by multiple males and therefore contained half‐sibs (Figure 3). We found a mean mating frequency of 2.7 fathers per matriline, with a range from one to five fathers. Additionally, we calculated the weighted average of fathers per matriline, called effective mating frequency (*m*_e_) that had a mean value of 1.8 and was smaller than the mean mating frequency. This shows that not all fathers sired an equal number of offspring per matriline. The main father per matriline sired 1 to 19 offspring (mean ± *SD* = 7 ± 4; total *n* offspring main fathers = 294), and all fathers together per matriline sired 1 to 29 offspring (mean ± *SD* = 11 ± 6; total *n* offspring all fathers = 439) (Figure 3).

Multiple paternity, and therefore higher genetic variability, could lead to differences in metamorphic traits within matrilines. We conducted paired *t* tests to compare the mean values of metamorphic traits of progeny within one matriline regarding single or multiple paternity. While the mean values of BCI and developmental time within matrilines were not changed by multiple paternity, we detected an increase in SVL with multiple paternity (Table 1). However, the differences were not supported by random subsampling of the matrilines. We conclude that the significant difference in mean SVL was due to a larger number of offspring in the all father dataset and does not represent a real effect based on multiple paternity.

**Table 1:** Comparison of metamorphic traits of offspring from main and all fathers within a matriline

Metamorphic trait	Mean	Subsampling (significant/not significant)	CV	Subsampling (significant/not significant)
SVL	*t* = −2.4663, *df* = 31, *p* = 0.01938, *d* = 0.44	3/10	*t* = −2.2049, *df* = 30, *p* = 0.03527, *d* = 0.4	1/10
BCI	*t* = 0.10494, *df* = 31, *p* = 0.9171, *d* = 0.02	0/10	*t* = −2.3403, *df* = 30, *p* = 0.02611, *d* = 0.42	3/10
Developmental time	*t* = 0.92399, *df* = 31, *p* = 0.362; *d* = 0.16	0/10	*t* = −4.6786, *df* = 30, *p* < 0.001, *d* = 0.84	10/10

Note

Given is a paired *t* test and Cohen's *d* as effect size for changes in mean values, changes in coefficient of variation (CV) and results from the subsampling to correct for different numbers of offspring from main father and all fathers.

To investigate whether variability of metamorphic traits was influenced by multiple paternity, we compared the coefficients of variation (CV) for both datasets.

The variability in all metamorphic traits was increased by multiple paternity when comparing main and all fathers (Table 1), but only developmental time was significantly more variable for offspring from all fathers (mean ± *SD*: 7.8 ± 3.0) than for offspring from main father (mean ± *SD*: 5.4 ± 1.8; Table 1) after random subsampling of metamorphs. This is supported by a large effect size (Cohens *d* = 0.84). Additionally, we detected a positive correlation of effective number of fathers and the CV in developmental time (Pearson correlation: *r* = 0.44, CI 0.15–0.66, *p* = 0.004), but not in the other metamorphic traits.

### 3.5 Differences of metamorphic traits between matrilines

**Figure 4:**
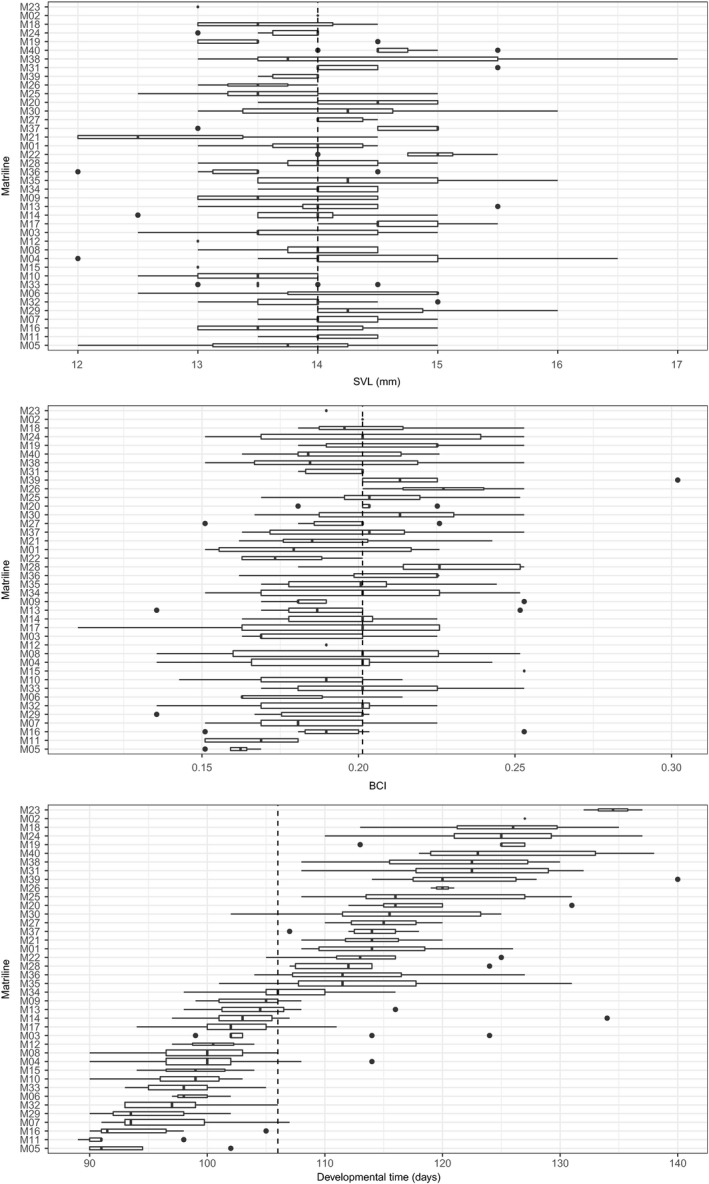
Snout‐vent length (SVL in mm), body condition index (BCI), and developmental time (days) of emigrated full‐sib metamorphs for each matriline. The dashed lines represent the overall median of size (14 mm), body condition (0.2013), and developmental time (107 days). Matrilines are ordered from short to long developmental time in each plot. The developmental time does not correlate with SVL or BCI. Box whisker plot: The box goes from 25th percentile to 75th percentile of the data. The line in the box indicates the median and the whiskers extending to the furthest data point that is within 1.5 times the box. Data points past the ends of the whiskers are considered outliers and are shown as black dots. The width of the box is proportional to the number of metamorphs per matriline

We tested if metamorphic traits differ between matrilines and therefore used the main father dataset of 295 individuals assigned to 40 matrilines (same broad genotype per matriline). The median SVL of metamorphs differed significantly between matrilines (Kruskal–Wallis rank sum test: χ^2^ = 84.89, *df* = 39, *p* < 0.0001), indicating a genetic or maternal effect. Overall, SVL of individuals ranged from 12 to 17 mm. The median of all individuals was 14 mm. The median SVL of the different matrilines ranged from 12.5 to 15 mm (Figure 4). After a post hoc Dunn test, we found that only two out of 780 comparisons between matrilines were significantly different concerning their SVL. Offspring from M17 had significantly bigger individuals (median 14.5 mm) than M10 and M33 (both median 13.5 mm; detailed results of Dunn test in Supporting Information Table S4). Nevertheless, in most comparisons, within matriline variability in SVL was higher than between matriline variability measured by the CV (range CV: 1.8–11.1, Table 2).

**Table 2:** Summary values for main father and all fathers’ offspring per matriline

Matriline	Number of metamorphs		Number of fathers		*m*_e_	SVL (mm)				Developmental time (days)				Body condition index			
	Main father	All fathers	Within clutch	All		Main father	CV	All fathers	CV	Main father	CV	All fathers	CV	Main father	CV	All fathers	CV
M1	6	7	1	2	1.3	13.9 ± 0.6	4.2	14.0 ± 0.6	4.1	115 ± 7	6.1	115 ± 6	5.6	0.19 ± 0.03	18.6	0.18 ± 0.03	17.1
M2	1	3	2^a^	3	3.0	14.0	NA	14.3 ± 0.6	4	127	NA	113 ± 17	4	0.20	NA	0.22	13
M3	9	11	1	2	1.4	13.9 ± 0.8	5.9	13.9 ± 0.9	6.2	106 ± 8	5.9	111 ± 14	6.2	0.18 ± 0.02	12.9	0.19 ± 0.03	16.4
M4	19	29	2	3	2.0	14.5 ± 1.1	7.4	14.3 ± 1.1	7.7	100 ± 6	6.1	100 ± 8	7.5	0.19 ± 0.03	14.9	0.19 ± 0.03	16.6
M5	4	14	2	4	3.2	13.6 ± 1.3	9.2	14.1 ± 1.1	7.5	94 ± 6	6.1	102 ± 13	13	0.16 ± 0.01	4.6	0.18 ± 0.03	14.3
M6	3	3	1	1	1.0	14.2 ± 1.4	10.2	14.2 ± 1.4	10.2	99 ± 3	2.7	99 ± 3	2.7	0.18 ± 0.03	16.5	0.18 ± 0.03	16.5
M7	14	17	2	2	1.4	14.2 ± 0.5	3.8	14.4 ± 0.7	4.6	96 ± 5	5	97 ± 5	5.1	0.19 ± 0.02	11.4	0.18 ± 0.02	10.6
M8	15	25	1	5	2.3	14.0 ± 0.5	3.3	14.2 ± 1.4	9.8	100 ± 5	4.9	107 ± 12	11.3	0.19 ± 0.04	18.6	0.19 ± 0.03	15.4
M9	5	6	2	2	1.4	13.7 ± 0.8	5.5	13.8 ± 0.7	5	104 ± 4	3.6	103 ± 4	3.4	0.19 ± 0.03	17.2	0.19 ± 0.03	18.6
M10	9	13	2	5	2.0	13.3 ± 0.6	4.6	13.4 ± 0.8	5.7	98 ± 4	4	99 ± 4	4.5	0.19 ± 0.02	12	0.19 ± 0.03	15.5
M11	5	9	2	3	2.3	14.1 ± 0.4	3	14.3 ± 0.9	6	92 ± 4	3.9	93 ± 5	5.3	0.17 ± 0.01	9	0.17 ± 0.02	11.3
M12	2	6	2	3	2.6	13.0	0	13.2 ± 0.5	3.9	101 ± 5	4.9	105 ± 13	12.8	0.19	0	0.20 ± 0.05	22.7
M13	8	8	1	1	1.0	14.1 ± 0.7	5.3	14.1 ± 0.7	5.3	105 ± 6	5.4	105 ± 6	5.4	0.19 ± 0.03	17.4	0.19 ± 0.03	17.4
M14	12	22	1	4	2.3	13.8 ± 0.8	5.5	13.9 ± 0.7	5	105 ± 10	9.1	103 ± 8	7.6	0.19 ± 0.02	10.6	0.19 ± 0.02	8.3
M15	2	2	1	1	1.0	13.0	0	13.0	0	99 ± 7	0	99 ± 7	0	0.25	0	0.25	0
M16	6	7	1	2	1.3	13.8 ± 0.9	6.4	13.7 ± 0.8	5.9	95 ± 6	6.2	96 ± 7	7.7	0.19 ± 0.03	17.2	0.19 ± 0.03	16.8
M17	13	16	1	3	1.5	14.7 ± 0.4	3	14.8 ± 0.4	2.8	102 ± 4	4.3	102 ± 4	3.9	0.19 ± 0.04	21.2	0.18 ± 0.04	20.1
M18	4	9	1	4	3.0	13.6 ± 0.8	5.5	13.9 ± 0.8	5.8	125 ± 9	7.4	123 ± 10	8.2	0.21 ± 0.03	15.7	0.20 ± 0.03	16.2
M19	5	7	1	3	1.8	13.5 ± 0.6	4.5	13.5 ± 06	4.3	123 ± 6	4.8	119 ± 9	7.9	0.21 ± 0.03	13.7	0.21 ± 0.03	12.4
M20	5	11	2^a^	2	2.0	14.4 ± 0.7	4.5	14.2 ± 0.7	4.8	119 ± 7	6.2	110 ± 12	10.6	0.20 ± 0.02	7.8	0.20 ± 0.02	11.8
M21	4	6	1	3	2.0	12.9 ± 1.2	9.2	13.3 ± 1.2	8.8	114 ± 5	4.4	116 ± 5	3.9	0.19 ± 0.03	17.9	0.19 ± 0.03	14.4
M22	4	4	2^a^	1	1.0	14.9 ± 0.6	4.2	14.9 ± 0.6	4.2	114 ± 8	7.2	114 ± 8	7.2	0.18 ± 0.02	10.5	0.18 v 0.02	10.5
M23	2	16	2	5	2.3	13.0	0	13.3 ± 0.8	5.7	135 ± 4	2.6	120 ± 8	6.7	0.19	0	0.21 ± 0.03	15
M24	10	11	1	2	1.2	13.8 ± 0.4	2.5	14.3 ± 1.6	11.2	125 ± 8	6.1	123 ± 10	7.8	0.20 ± 0.04	20.3	0.20 ± 0.04	19.8
M25	7	13	1	4	2.5	13.6 ± 0.8	5.9	13.9 ± 0.8	5.5	119 ± 9	7.6	114 ± 12	10.5	0.21 ± 0.03	12.7	0.20 ± 0.03	16.1
M26	2	6	1	3	2.6	13.5 ± 0.7	5.2	13.8 ± 0.6	4.5	120 ± 1	1.2	123 ± 6	4.7	0.23 ± 0.04	16.1	0.19 ± 0.05	25.5
M27	6	14	1	4	2.6	14.2 ± 0.3	1.8	14.0 ± 0.6	4.2	115 ± 4	3.4	110 ± 8	7.4	0.19 ± 0.03	13.1	0.20 ± 0.03	13.9
M28	11	13	NA	3	1.4	14.1 ± 0.6	4	13.9 ± 0.8	5.5	112 ± 5	4.6	111 ± 9	8.5	0.23 ± 0.02	10.7	0.23 ± 0.02	10.2
M29	6	6	NA	1	1.0	14.6 ± 0.8	5.5	14.6 ± 0.8	5.5	95 ± 5	4.9	95 ± 5	4.9	0.18 ± 0.03	15.1	0.18 ± 0.03	15.1
M30	8	10	NA	2	1.5	14.2 ± 1.0	7.3	14.1 ± 1.0	7	116 ± 8	7.2	114 ± 12	10.1	0.21 ± 0.03	14.7	0.21 ± 0.03	14.3
M31	8	11	NA	4	1.8	14.3 ± 0.5	3.7	14.2 ± 0.5	3.6	122 ± 8	6.8	117 ± 12	10.4	0.19 ± 0.01	5.2	0.19 ± 0.02	9.1
M32	13	13	NA	1	1.0	13.9 ± 0.5	3.7	13.9 ± 0.5	3.7	97 ± 4	3.9	97 ± 4	3.9	0.19 ± 0.03	16.2	0.19 ± 0.03	16.2
M33	9	11	NA	3	1.5	13.6 ± 0.5	3.4	13.6 ± 0.5	3.5	98 ± 4	4	101 ± 9	9.2	0.20 ± 0.03	14.4	0.21 ± 0.03	14.7
M34	13	14	NA	2	1.2	14.1 ± 0.3	2.5	14.1 ± 0.3	2.4	107 ± 5	5.1	106 ± 5	5	0.20 ± 0.03	15.9	0.20 ± 0.03	15.2
M35	12	22	NA	4	2.3	14.3 ± 0.8	5.9	14.5 ± 1.1	7.2	114 ± 8	7.2	113 ± 7	6	0.20 ± 0.02	12.6	0.20 ± 0.03	15.4
M36	6	11	NA	3	2.3	13.3 ± 0.8	6.1	13.2 ± 0.6	4.6	113 ± 8	7.5	111 ± 7	6.7	0.21 v 0.03	13	0.21 ± 0.04	18.5
M37	7	11	NA	3	2.1	14.6 ± 0.7	5	14.5 ± 0.6	4.4	114 ± 4	3.2	115 ± 6	5.1	0.20 ± 0.03	16.7	0.20 ± 0.03	15.3
M38	6	6	NA	1	1.0	14.5 ± 1.6	11.1	14.5 ± 1.6	11.1	121 ± 9	7.1	121 ± 9	7.1	0.19 ± 0.04	20.3	0.19 ± 0.04	20.3
M39	6	6	NA	1	1.0	13.8 ± 0.3	1.9	13.8 ± 0.3	1.9	123 ± 9	7.7	123 ± 9	7.7	0.23 ± 0.04	12.5	0.23 ± 0.04	12.5
M40	7	10	NA	3	1.9	14.6 ± 0.5	3.3	14.4 ± 0.6	3.9	126 ± 8	6.7	127 ± 7	5.7	0.19 ± 0.02	12.5	0.19 ± 0.02	11.2

Note

Shown are the number of metamorphs (for main and all fathers), the number of fathers that sired offspring (found within the clutch and all offspring), the effective mating frequency (*m*_e_), the size of metamorphs (SVL in mm), developmental time (days), and the body condition index. Metamorphic traits are given as mean ±SD and the corresponding coefficient of variation (CV). NA: number of father genotypes in clutch is unknown, because those metamorphs were clustered without clutch samples.

a Clutch samples with more than one father genotype, but not for each father genotype metamorphs were found.

We found no significant difference between the median BCI of matrilines (Kruskal–Wallis rank sum test: χ^2^ = 51.89, *df* = 39, *p* = 0.08107). Overall, BCI of individuals ranged from 0.11 to 0.30 with a median of 0.20. The median BCI of the different matrilines ranged from 0.16 to 0.23 (Figure 4). BCI was less variable between matrilines, but showed a high variability within matrilines (range CV: 4.6–21.2, Table 2).

The developmental time differed significantly between matrilines (Kruskal–Wallis rank sum test: χ^2^ = 226.21, *df* = 39, *p* < 0.0001), indicating a genetic or maternal effect. Overall, developmental time of individuals ranged from 89 to 140 days, with a median of 106 days. The median developmental time of the different matrilines ranged from 91 to 135 days (Figure 4). After a post hoc Dunn test, we found that 267 of 780 comparisons between matrilines were significantly different concerning their developmental time. For example, offspring of M11 (median 91 days) had a significantly shorter developmental time than offspring of M23 (median 135 days; results of Dunn test in Supporting Information Table S5). In fact, all metamorphosing offspring of M11 had left before offspring of M23 had started to leave the pond. Developmental time showed the highest variability between matrilines, but also high within‐matriline variation (range CV: 1.2–9.1, Table 2).

### 3.6 Influence of number of offspring and number of fathers on metamorphic traits

Life‐history strategies could differ between mothers, where some invest in a small number of eggs with higher amount of resources than average, or they invest in a large number of eggs with a lower amount of resources than average. The amount of resources in the egg should be positively correlated with the body size at metamorphosis.

**Figure 5:**
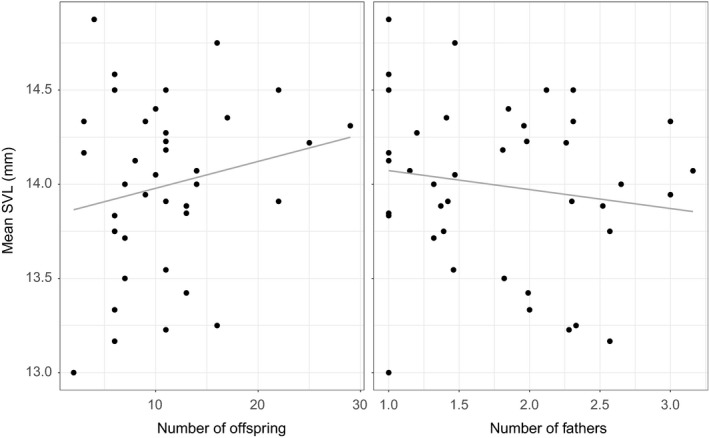
Mean snout‐vent length (SVL in mm) of metamorphs per matriline (*n* = 40) in relation to the number of offspring per matriline (left) and number of fathers (right). The gray line is the linear model of number of offspring and number of fathers to mean SVL; *F*(3,36) = 2.909, *p* = 0.04771

The linear model of mean SVL per matriline, *F*(3,36) = 2.909, *p* = 0.04771, *R* ^2^ = 0.13, suggested that developmental time did not influence SVL (*β* = 0.0086, *p* = 0.2592), but that number and size of offspring was positively related (*β* = 0.0367, *p* = 0.0125). This indicates that some matrilines had more metamorphosing offspring with larger SVLs (Figure 5). Interestingly, the number of fathers had a negative influence on size (*β* = −0.1817, *p* = 0.0125; Figure 5).

Additionally, we ran the same model with effective number of fathers to account for the different proportion of offspring sired; however, none of the variables influenced SVL (model in Appendix S2). Nevertheless, the trend in the data was the same, with number of offspring having a positive influence and effective number of fathers a negative influence.

The BCI could not be fitted to a linear model, as assumptions were not met. Instead, we used a GAM. Neither number of offspring, nor number of fathers (or effective number of fathers) had an influence on the mean BCI of matrilines, when included as smoothed terms. The developmental time showed a positive albeit not significant linear trend (*β* = 0.0005, *p* = 0.095), indicating a higher BCI per matriline with longer pond development. The summary of the GAM can be found in the Appendix (S3, Supporting Information Figure S5).

## 4 DISCUSSION

Our hypotheses were that high variability in metamorphic traits between offspring from different matrilines developing in the same environment should be influenced by maternal and paternal genetic effects. Additionally, multiple paternity should increase genetic variability of offspring within matrilines, therefore increasing variability in metamorphic traits of *R*. *temporaria*. We found low variability between matrilines in size and body condition, but high variability in developmental time in the same environment, potentially due to genetic effects. In addition, multiple paternity seems to be very common and increased the variability of developmental time, but not the variability of other metamorphic traits. Additive genetic effects of fathers seem to act on developmental time, because we found a positive relationship of the effective number of fathers and the variability in developmental time within single matrilines. Additionally, the number of metamorphosed offspring differed between matrilines, with some having a higher number of progeny with larger SVL, indicating that some *R. temporaria* females reproduce more successfully than others in the same environment.

### 4.1 Overall emigration pattern and relationship of size and BCI with developmental time

The size and BCI of all emerging metamorphs was only marginally influenced by developmental time and did not show a linear relationship. Consequently, there was almost no difference in size or BCI of metamorphs with a short or a long developmental time. These findings contrast with former models on amphibian metamorphosis (e.g., Wilbur & Collins, 1973). However, environmental stress, such as food shortage, decreasing water level, and predation or density effects (exploitative or interference competition) in a natural environment, could lead to a negative relationship of size and age of metamorphosis (Laurila et al., 2001; Merilä, Laurila, Laugen, Räsänen, & Pahkala, 2000; Relyea, 2007; Wong, Griffiths, & Beebee, 2000). The multiple biotic and abiotic influences acting in parallel in our natural system may have led to trade‐offs between growth and development (Laugen et al., 2003; Loman, 2016) and could counteract potential underlying intrinsic genetic effect (Conover & Schultz, 1995). Former studies on the same natural population showed similar patterns (Grözinger et al., 2018, 2014), where environmental factors alone could not explain the variation in observed metamorphic traits.

### 4.2 Differences of metamorphic traits between matrilines of *R. temporaria*

We assumed that the variability observed in former studies (Grözinger et al., 2018, 2014) might be due to differences in resource allocation or intrinsic genetic effects within single matrilines. Indeed, when we assigned metamorphs to their respective matrilines, we detected differences in metamorphic traits among offspring from different mothers. We observed the most profound differences in developmental time, where metamorphs from fast developing matrilines left the pond before individuals from slow developing matrilines even started emigration. Even if the breeding spanned over approximately 12 days, we think that the first clutches could not experience a developmental advantage due to unfavorable weather conditions (Loman, 2002). Therefore, priority effects (Eitam, Blaustein, & Mangel, 2005; Wong et al., 2000) should have limited, if any, effect on developmental time. However, there were fewer differences in SVL and BCI than in developmental time. When exploitative competition occurs and tadpoles of different size classes are competing for limited resources, an intermediate size could be favored (van Buskirk, Cereghetti, & Hess, 2017). This would counteract intrinsic genetic effects concerning growth rate and therefore developmental time.

### 4.3 Multiple paternity and differences in metamorphic traits

Multiple paternity is frequently observed in anuran species and was detected in three European explosive breeders (*R. temporaria*: Laurila & Seppä, 1998; *R. arvalis*: Knopp & Merilä, 2009; *R*. *dalmatina*: Lodé & Lesbarréres, 2004). The advantage of a polyandrous or lek mating system is higher genetic diversity of progeny from a mother, which leads to increased survival probabilities and fitness of offspring (Jennions & Petrie, 2000). This ensures that at least some offspring will survive in unpredictable environments (Yasui, 1998). In our study, the different fathers did not sire an equal number of offspring per clutch. This effect is due to the external fertilization process. The sperm is released simultaneously with the eggs of the female, and fertilization can take place within minutes in large breeding aggregations (Savage, 1961). However, even a few seconds after egg deposition clutch piracy can occur and another “sneaky” male can fertilize the remaining unfertilized eggs (Vieites et al., 2004), which results in different numbers of offspring sired by several males. Therefore, multiple paternity is unlikely an effect of active mate choice of females within the breeding aggregation (Dittrich et al., 2018). The advantage of increased genetic variability among offspring within a clutch will increase the chance that some individuals will survive. Therefore, polygamous mating systems can be seen as a bet‐hedging strategy to decrease variability of survivorship between years, especially when the environment is unpredictable and multiple paternity has no additional cost (Yasui, 2001).

We show that multiple paternity leads to higher variability of developmental time in metamorphs within a matriline and on average, longer period of emigration from offspring of the respective female. Additionally, we showed that the effective number of fathers increases this variability.

The effect of higher variability was not detected for size and body condition of offspring, which indicates that these metamorphic traits are probably more influenced by other parameters, such as maternal provisioning or environmental cues. Even when intrinsic effects were found under controlled laboratory conditions, these effects could not be detected under field conditions due to countergradient variation (Laugen et al., 2003). Additionally, it was shown before that those traits, which are important indicators of future fitness, should not be affected by additive genetic variance (Berven & Gill, 1983). Therefore, SVL and BCI seem to be more important proxies for future fitness of metamorphs in our system than developmental time.

### 4.4 Influence of number of offspring and number of fathers on metamorphic traits

Due to an inverse relationship between reproductive investment per offspring and number of offspring (Charnov & Ernest, 2006; Smith & Fretwell, 1974), individual females have to trade‐off number and size of progeny. Therefore, we examined a possible trade‐off between the number of successfully metamorphosed offspring per matriline (after natural selection) and their size/body condition and developmental time. We found a positive relationship between the number of metamorphosed offspring and the metamorphic size, but no influence of developmental time, which seems to be in contrast to the known models. Most models of optimal timing and size of metamorphosis are based on maximizing growth rate, to minimize mortality risk in the aquatic and terrestrial habitat and/or taking time constraints into consideration (Rowe & Ludwig, 1991; Rudolf & Rödel, 2007; Wilbur & Collins, 1973). A rapid growth could be favored under time constraints or/and if a minimum size has to be reached for niche transition, or if predation is size dependent (Arendt, 1997). We did not monitor predator densities in the pond, but we observed alpine newts (*Ichthyosaurus alpestris*) sitting under freshly laid clutches, feeding on the embryos. Therefore, we think that predation pressure in the early development could have been high, which could lead to smaller size and lower size variability of progeny (van Buskirk & Relyea, 1998). Additionally, high tadpole densities lead to slower growth and smaller size at metamorphosis (Loman, 2004). In natural populations, densities are high in the beginning (large number of eggs from clutches) and decrease over time due to predation and high mortality in the larval stage (Wilbur, 1980). Therefore, a longer developmental time can be beneficial when tadpoles and predator densities are decreasing over time and single individuals attain more resources for growth in the aquatic stage, which could promote better/higher BCI at metamorphosis. This would be supported by the positive trend we found in BCI over time, where a longer development leads to higher BCI and therefore higher survival probability (Scott et al., 2007). We could not detect any influence of number of fathers or number of successfully developing metamorphs on BCI.

Still, we found a higher number of offspring with bigger body size in single matrilines. In anurans, the egg size correlates strongly with body size (Cummins, 1986) and has a negative relationship with egg number (Jørgensen, 1981). The egg size influences growth and developmental rates, but is not per se responsible for differences in metamorphic size (Loman, 2002). We cannot rule out the possibility of different age/size classes of females and therefore different provisioning or number of eggs per female. However, body size differences have been small in our study and could be canalized, as this trait is highly fitness relevant (Berven & Gill, 1983) in our study pond. The heritability of traits could differ dependent on different selection pressures in the environment, with lower heritability in canalized traits (Berven & Gill, 1983). Therefore, developmental time seems to have a higher heritability because variability in this trait is influenced by additive effects of fathers, while body size and condition were only marginally affected by multiple paternity.

## 5 SUMMARY

In our study, we could show that metamorphic traits differ between matrilines in the same environment, which indicates that there are underlying intrinsic genetic effects from the parents. However, SVL and BCI differed only marginally between matrilines, an indication for strong environmental effects that are counteracting the intrinsic growth rates. These environmental effects could be predation pressure and the amount of food resources, as well as temperature and desiccation risk. We show that multiple paternity is very common in this *R. temporaria* population and increases the variability in metamorphic traits, especially in developmental time of offspring from the same matriline. This increase seems to be due to additive genetic effects of multiple fathers on developmental time. These findings suggest that SVL and BCI are more influenced by environmental factors.

## CONFLICT OF INTEREST

None declared.

## AUTHOR CONTRIBUTIONS

MOR and HF designed the research; JH performed the research; CD and JH analyzed the data; CD, JH, MOR, and HF wrote the manuscript.

## Data Availability

Data and R‐source code to reproduce the analysis and figures are archived at Dryad Digital Repository and are accessible with the following link: https://doi.org/10.5061/dryad.mf4h560.
