# Supplementary material for: Matriline effects on metamorphic traits in a natural system in the European common frog (Rana temporaria)
Source: Ecol Evol. 2019 Feb 21;9(6):3075–88. doi: 10.1002/ece3.4811 (PMC6434577; doi:10.1002/ece3.4811)
Supplement: Supplementary file 1 [file ECE3-9-3075-s001.doc]

**Supplemental Information for:**

**Matriline effects on metamorphic traits in a natural system in the European common frog (*Rana temporaria)***

Carolin Dittrich, Juliane Huster, Mark-Oliver Rödel & Heike Feldhaar

**Table of content**

Figure S1: Picture of fenced study pond 2

Figure S2: daily water temperatures and amount of rain during the study period 3

Table S1: Details on PCR mix 4

Table S2: Details on primer pairs 5

Figure S3: GAM model, Relationship of developmental time in days and SVL in mm 6

Figure S4: GAM model, Relationship of developmental time in days and BCI 6

Table S3: Number and effective number of alleles per locus 7

Appendix S1 Methodological details on sibship analysis 7

Table S4. Results of Dunn-test on SVL 8

Table S5. Results of Dunn-test on developmental time 25

Appendix S2 Additional linear model of effective mating frequency and body size 42

Appendix S3 Summary GAM BCI and developmental time 43

Figure S5 GAM developmental time and BCI 43

**
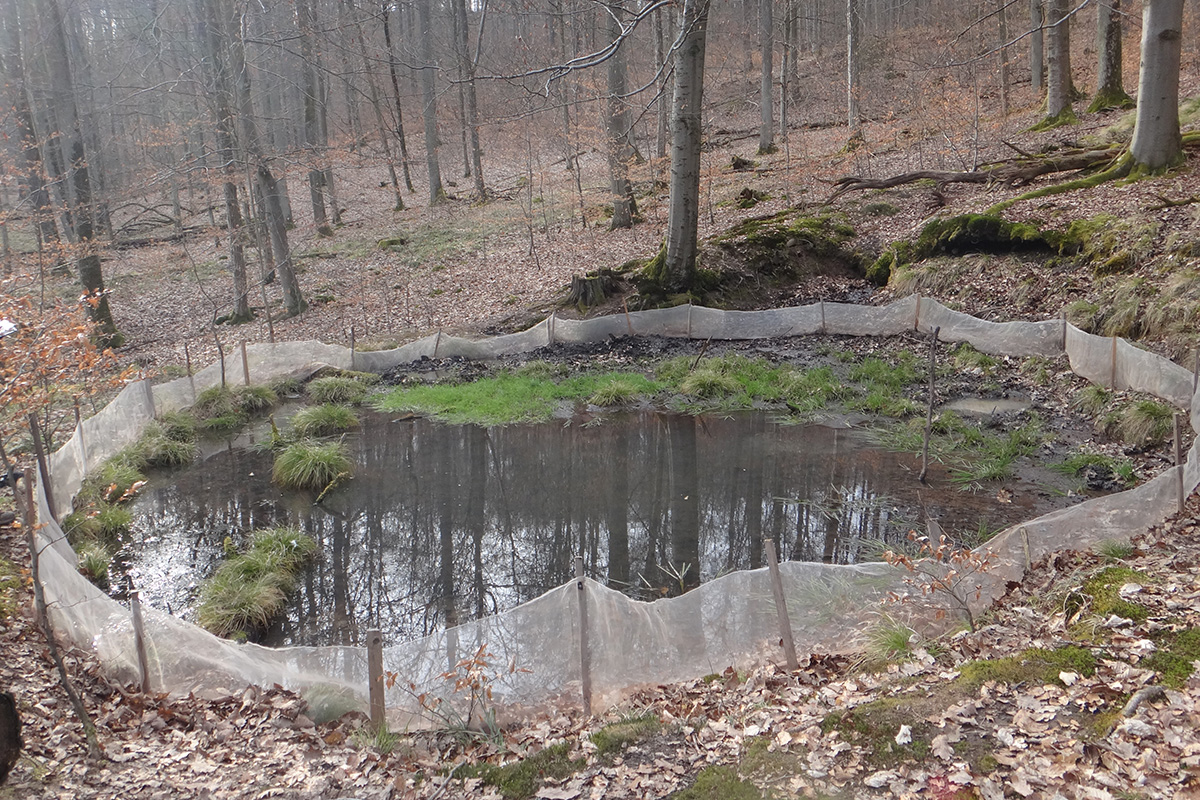
**

**Figure S1** Picture of the fenced study pond (GPS: N 49° 54' 53.04348 E 10° 32' 52.974456, WGS84)

**
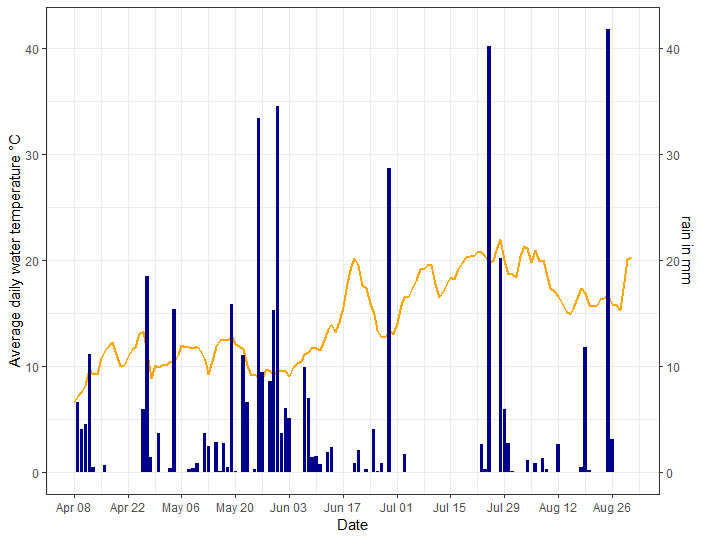
**

**Figure S2** The orange line gives the average daily water temperature (°C) in the pond over the whole period from spawning to last metamorphs emerging (April 08^th^ – August 31^st^ 2013). Temperature was measured with a Thermochron iButton© every 3 hours and mean calculated per day. The blue bars represent the daily amount of rain in mm, measured at a local weather station 2.5 km away from the study pond.

**Table S1 PCR mastermix per sample for amplifying microsatellite DNA.**

| **component** | **company** | **information** | **volume [µl]** |
| --- | --- | --- | --- |
| HPLC-H20 | VWR |  | 7.75 |
| 10x reaction buffer Y | Peqlab | 10 x reaction buffer Y („high yields”): 200 mM Tris-HCL (pH 8.55), 160 mM  (NH4)2SO4, 0.1 % Tween 20, 20 mM  MgCl2 | 1.25 |
| dNTP | Genaxxion BioScience | pH 7.5, 2 mM | 1 |
| Forward primer | Metabion | stock solution: 100 pmol/µl, 1:10 diluted | 0.2 |
| Reverse primer | Metabion | stock solution: 100 pmol/µl, 1:10 diluted | 0.2 |
| Taq | Peqlab | 5 U/µl | 0.1 |
| + 2 µl template DNA | | | |

**Table S2 Primer sequences and characteristics of seven microsatellite loci in *R. temporaria* (Matsuba & Merilä, 2009). Primers were labelled at 5’ end.**

| **locus** | **core motif** | **repeats** | **temp. [°C]** | **primer sequence (5‘-3‘)** | **label** | **allele range [bp]** |
| --- | --- | --- | --- | --- | --- | --- |
| BFG046 | TG | 29 | 55 | F-GGAGCATTGGAAAGAACACAAT  R-GTTTGCGTAGGTAGGCCATGT | -  IRD700 | 190-280 |
| BFG090 | CTAT | 16 | 55 | F-CCTGGGCATTTACCTCATACAT  R-TCCCTATACGCATTCCAAAATC | -  IRD700 | 185-320 |
| BFG099 | ACTC | 14 | 55 | F-CAGTAAGGAATGGATACTAAGC R-TCCAGTGTAGCATAACAGAGT | -  IRD700 | 90-180 |
| BFG203 | TTCT*2 | (23)(4) | 55 | F-ATATCTTCCCTTTCCCCTTTCA  R-GCAACAGAGCAGACAGAGAGAG | IRD800  - | 165-234 |
| BFG237 | GATA | 19 | 55 | F-GGATTCTACGGATCTTTGGACA  R-CCTTCCATTCTGTTTGTTAGGC | IRD800  - | 200-300 |
| BFG242 | GATA | 28 | 55 | F-GGACCTCAATGGCCTTATCAGT  R-CAATCAACTGGCCCCACC | IRD700  - | 210-290 |
| BFG250 | GATA | 20 | 55 | F-CCTGTTAGAGAAGCCGATCATT  R-TTGGACTGGAAGTATTGGGAGT | IRD800  - | 220-338 |

temp. = annealing temperature; primer sequences: F = forward primer, R = reverse primer; IRD-700/800 = fluorescence dye label


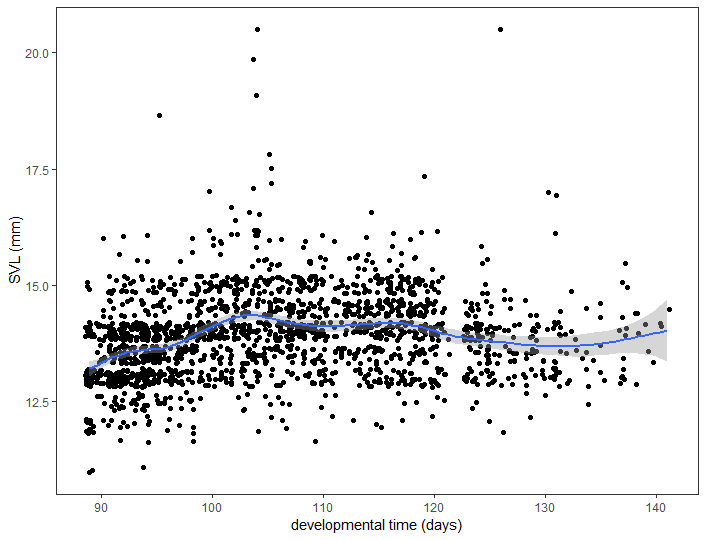

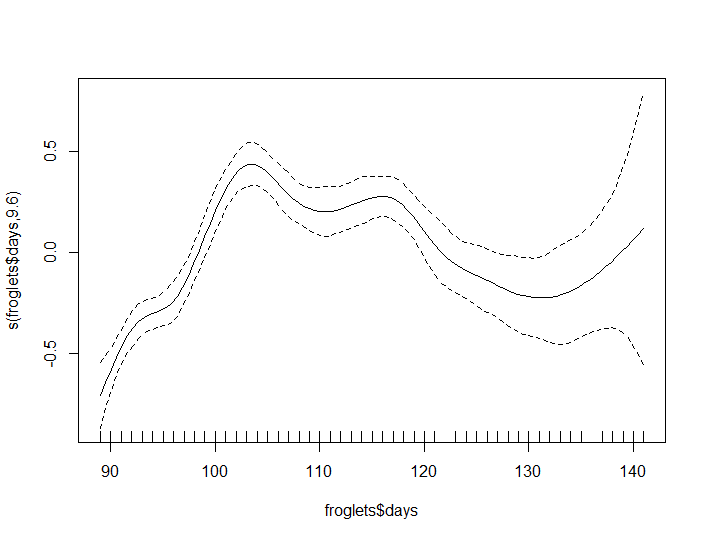


**Figures S3** Relationship of developmental time in days and SVL in mm of *Rana temporaria* metamorphs (*n* = 1943). Left: Datapoints are jittered to avoid overplotting. Blue line is the GAM (method=REML, cubic regression spline, 15 knots). Deviance explained (R^2^) is 12.4% and time has a significant effect on size at metamorphosis (*p* < 0.001), but not a linear one. Right: Model output for the relationship of developmental time and body size.


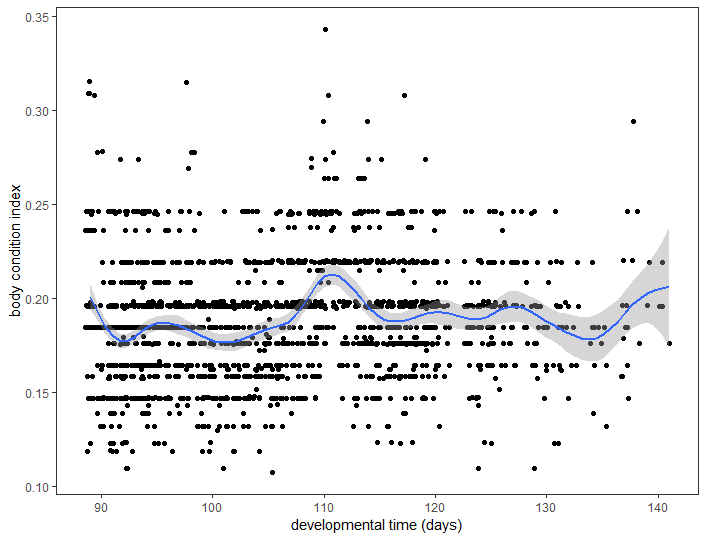

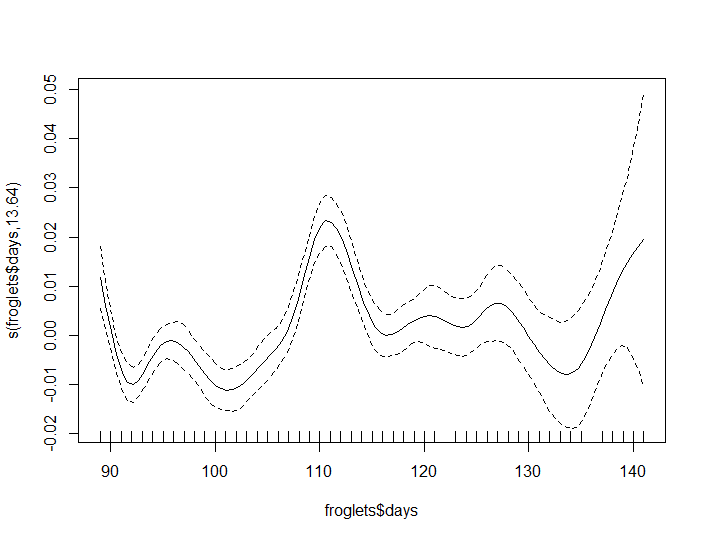


**Figures S4** Relationship of developmental time in days and BCI in g of *Rana temporaria* metamorphs (*n* = 1943). Left: Datapoints are jittered to avoid overplotting. Blue line is the GAM (method=REML, cubic regression spline, 20 knots). Deviance explained (R^2^) by the model is 8.5% and time has a significant effect on BCI at metamorphosis (*p* < 0.001), but not a linear one. Right: Model output for the relationship of developmental time and body condition index.

**Table S3 Number and effective number of alleles per locus (loci derived from Matsuba & Merilä, 2009), observed and expected heterozygosity.**

| **Locus** | **Number of alleles** | **Effective number of alleles (Ae)** | **Observed heterozygosity (H_O_)** | **Expected heterozygosity (H_E_)** |
| --- | --- | --- | --- | --- |
| **BFG046** | 25 | 3.6 | 0.61 | 0.72 |
| **BFG090** | 23 | 13.2 | 0.92 | 0.92 |
| **BFG099** | 16 | 5.2 | 0.83 | 0.82 |
| **BFG203** | 14 | 5.2 | 0.83 | 0.81 |
| **BFG237** | 15 | 9.9 | 0.90 | 0.90 |
| **BFG242** | 15 | 9.6 | 0.79 | 0.90 |
| **BFG250** | 20 | 7.8 | 0.88 | 0.87 |

Appendix S1. **Methodological details on sibship analysis**

Clutch sample: From the total number of 228 embryos, 177 were successfully genotyped (five loci: 14, six loci: 49, seven loci: 114). In eleven clutch samples we found two father genotypes. For three of these multiple paternity clutch samples, we detected offspring of only one of these fathers when assigning metamorphs to sibships.

Metamorph samples: For 28 individuals the amplification of microsatellite loci failed completely. The amplification for at least five out of seven loci was successful in 60% of the individuals.

There was no locus indicating large allele dropout and only one locus (Locus BFG242) showed stuttering that could lead to scoring errors. Therefore, the scoring of this locus was checked manually again. Two loci (Locus BFG046 and Locus BFG242) showed more homozygotes than expected and may contain null alleles. However, the presence of related offspring and uneven numbers of offspring per matriline may cause such deviation from HWE (Wigginton, Cutler, & Abecasis, 2005). Hence, the loci were not excluded from further analysis, because the assignment of metamorphs to sibship groups had lower probabilities without the loci deviating from HWE.

Wigginton, J. E., Cutler, D. J., & Abecasis, G. R. (2005). A note on exact tests of Hardy-Weinberg equilibrium. *The American Journal of Human Genetics, 76*, 887-893. https://doi.org/https://doi.org/10.1086/429864

**Table S4** Results of a Dunn test with fdr correction for multiple testing for snout-vent length of metamorphs between matrilines. Given are the respective comparisons of matriline number (M1-M40), the z-value, the unadjusted p-value (res.P.unadj) and the adjusted p-value with fdr correction (res.P.adj). Comparisons are sorted from smallest to largest adjusted p-value.

| **res.Comparison** | **res.Z** | **res.P.unadj** | **res.P.adj** |
| --- | --- | --- | --- |
| M10 - M17 | -4.308555544 | 1.64E-05 | 0.012817288 |
| M17 - M33 | 3.911072452 | 9.19E-05 | 0.035836008 |
| M17 - M36 | 3.665007242 | 0.000247332 | 0.064306212 |
| M10 - M40 | -3.565988404 | 0.000362487 | 0.070685043 |
| M17 - M21 | 3.489585729 | 0.00048377 | 0.075468098 |
| M17 - M25 | 3.15670079 | 0.00159565 | 0.082973793 |
| M17 - M24 | 3.116851142 | 0.001827938 | 0.083870118 |
| M33 - M37 | -3.067166659 | 0.002160983 | 0.084278351 |
| M10 - M37 | -3.409183391 | 0.000651577 | 0.08470496 |
| M04 - M10 | 3.296183959 | 0.000980078 | 0.084940108 |
| M12 - M17 | -3.165146672 | 0.001550048 | 0.086359813 |
| M36 - M40 | -3.123278304 | 0.001788485 | 0.087188666 |
| M14 - M17 | -3.069153682 | 0.002146661 | 0.088126096 |
| M12 - M40 | -2.909607191 | 0.003618833 | 0.088209047 |
| M17 - M32 | 3.223826856 | 0.001264899 | 0.089692806 |
| M15 - M40 | -2.909607191 | 0.003618833 | 0.0910545 |
| M22 - M33 | 3.023888974 | 0.00249548 | 0.092689258 |
| M21 - M40 | -3.069675896 | 0.002142912 | 0.09285951 |
| M15 - M17 | -3.165146672 | 0.001550048 | 0.093002876 |
| M36 - M37 | -2.981240932 | 0.002870828 | 0.093301921 |
| M23 - M40 | -2.909607191 | 0.003618833 | 0.09408965 |
| M22 - M36 | 2.989995439 | 0.002789816 | 0.094611156 |
| M17 - M19 | 3.297965236 | 0.000973882 | 0.094953486 |
| M10 - M29 | -2.913487248 | 0.003574164 | 0.096132696 |
| M04 - M33 | 2.870236425 | 0.00410165 | 0.096948089 |
| M21 - M22 | -2.993074548 | 0.002761823 | 0.097919172 |
| M33 - M40 | -3.223971672 | 0.001264259 | 0.098612206 |
| M12 - M22 | -2.91593965 | 0.003546191 | 0.098786746 |
| M17 - M23 | 3.165146672 | 0.001550048 | 0.100753115 |
| M21 - M37 | -2.943599922 | 0.003244191 | 0.101218753 |
| M15 - M22 | -2.91593965 | 0.003546191 | 0.102445514 |
| M19 - M40 | -2.842296351 | 0.004478984 | 0.102753153 |
| M10 - M22 | -3.310713737 | 0.000930584 | 0.103693594 |
| M12 - M37 | -2.811049068 | 0.004938025 | 0.104098915 |
| M22 - M23 | 2.91593965 | 0.003546191 | 0.106385726 |
| M15 - M37 | -2.811049068 | 0.004938025 | 0.106990552 |
| M23 - M37 | -2.811049068 | 0.004938025 | 0.110047425 |
| M19 - M22 | -2.767775798 | 0.005644027 | 0.115851077 |
| M07 - M10 | 2.738616043 | 0.006169838 | 0.123396765 |
| M04 - M36 | 2.721261783 | 0.006503324 | 0.126814815 |
| M19 - M37 | -2.707340084 | 0.006782474 | 0.129032429 |
| M16 - M17 | -2.682299257 | 0.007311802 | 0.135790608 |
| M04 - M21 | 2.655308281 | 0.007923596 | 0.13734233 |
| M03 - M17 | -2.669322947 | 0.007600433 | 0.137868324 |
| M09 - M17 | -2.644418985 | 0.008183129 | 0.13875741 |
| M10 - M31 | -2.658983366 | 0.007837683 | 0.138940749 |
| M25 - M40 | -2.635358501 | 0.008404846 | 0.139484679 |
| M17 - M18 | 2.601011338 | 0.009294938 | 0.145001036 |
| M04 - M12 | 2.514947417 | 0.011905014 | 0.145092356 |
| M12 - M29 | -2.536849647 | 0.011185498 | 0.145411477 |
| M08 - M17 | -2.584957608 | 0.009739097 | 0.146086456 |
| M10 - M35 | -2.603947985 | 0.009215674 | 0.146698487 |
| M21 - M29 | -2.575494514 | 0.010009686 | 0.147312362 |
| M04 - M15 | 2.514947417 | 0.011905014 | 0.147395409 |
| M15 - M29 | -2.536849647 | 0.011185498 | 0.147876078 |
| M22 - M25 | 2.554408871 | 0.010636827 | 0.148155804 |
| M29 - M33 | 2.586456702 | 0.009696835 | 0.148304539 |
| M10 - M20 | -2.566499831 | 0.010273068 | 0.148388753 |
| M17 - M39 | 2.605144498 | 0.009183552 | 0.149232721 |
| M32 - M40 | -2.545317272 | 0.010917849 | 0.149402142 |
| M04 - M23 | 2.514947417 | 0.011905014 | 0.149772754 |
| M29 - M36 | 2.556654229 | 0.010568421 | 0.149879425 |
| M23 - M29 | -2.536849647 | 0.011185498 | 0.150425666 |
| M24 - M40 | -2.515782506 | 0.011876845 | 0.151867854 |
| M25 - M37 | -2.487521317 | 0.012863674 | 0.154364093 |
| M14 - M40 | -2.433623828 | 0.014948521 | 0.176664344 |
| M22 - M24 | 2.420848478 | 0.01548433 | 0.177614374 |
| M22 - M32 | 2.42345824 | 0.015373521 | 0.178975316 |
| M17 - M28 | 2.24991653 | 0.024454244 | 0.190743107 |
| M04 - M19 | 2.389445453 | 0.016873829 | 0.190747636 |
| M16 - M40 | -2.251498201 | 0.024354001 | 0.191880006 |
| M09 - M40 | -2.254943204 | 0.024136894 | 0.192109973 |
| M16 - M22 | -2.238615485 | 0.025180946 | 0.192560174 |
| M09 - M22 | -2.255091939 | 0.024127559 | 0.194015419 |
| M17 - M26 | 2.238985055 | 0.025156889 | 0.194280922 |
| M32 - M37 | -2.376756948 | 0.017465592 | 0.1946166 |
| M07 - M12 | 2.256628668 | 0.024031287 | 0.195254211 |
| M12 - M35 | -2.204899211 | 0.027461168 | 0.19651111 |
| M31 - M33 | 2.304268935 | 0.021207552 | 0.196927265 |
| M12 - M20 | -2.351387188 | 0.01870356 | 0.197145634 |
| M07 - M15 | 2.256628668 | 0.024031287 | 0.197309518 |
| M10 - M34 | -2.307629925 | 0.021019729 | 0.197534799 |
| M12 - M31 | -2.312029907 | 0.020776035 | 0.197625701 |
| M15 - M35 | -2.204899211 | 0.027461168 | 0.198330657 |
| M07 - M23 | 2.256628668 | 0.024031287 | 0.199408556 |
| M15 - M20 | -2.351387188 | 0.01870356 | 0.199846259 |
| M15 - M31 | -2.312029907 | 0.020776035 | 0.200065525 |
| M21 - M35 | -2.208650717 | 0.027198945 | 0.200143179 |
| M23 - M35 | -2.204899211 | 0.027461168 | 0.200184215 |
| M07 - M33 | 2.335195115 | 0.01953323 | 0.200472626 |
| M01 - M17 | -2.211249149 | 0.027018589 | 0.200709515 |
| M10 - M27 | -2.192879243 | 0.028316081 | 0.200786753 |
| M20 - M33 | 2.257485011 | 0.023977785 | 0.201104004 |
| M14 - M22 | -2.337958755 | 0.019389388 | 0.201649634 |
| M17 - M34 | 2.212106504 | 0.026959307 | 0.202194801 |
| M18 - M40 | -2.259089605 | 0.023877812 | 0.202442316 |
| M23 - M31 | -2.312029907 | 0.020776035 | 0.202566344 |
| M20 - M23 | 2.351387188 | 0.01870356 | 0.202621902 |
| M19 - M29 | -2.31653516 | 0.020529067 | 0.202692054 |
| M35 - M36 | 2.177551066 | 0.029439476 | 0.203210542 |
| M24 - M37 | -2.355430566 | 0.018501254 | 0.203253216 |
| M18 - M22 | -2.274566596 | 0.022931942 | 0.203260394 |
| M20 - M36 | 2.265895085 | 0.023457808 | 0.203300999 |
| M07 - M21 | 2.287686666 | 0.022155775 | 0.203311816 |
| M33 - M35 | -2.21307172 | 0.026892701 | 0.203653467 |
| M31 - M36 | 2.28228741 | 0.022472377 | 0.203819237 |
| M22 - M39 | 2.179622844 | 0.02928543 | 0.203952102 |
| M39 - M40 | -2.183052656 | 0.029031933 | 0.204008175 |
| M20 - M21 | 2.323210773 | 0.020167832 | 0.204297522 |
| M05 - M17 | -2.259251957 | 0.023867717 | 0.204580428 |
| M14 - M37 | -2.267469177 | 0.02336158 | 0.204741935 |
| M07 - M36 | 2.276066793 | 0.022842012 | 0.204790454 |
| M21 - M31 | -2.317161168 | 0.020494954 | 0.20494954 |
| M03 - M40 | -2.155500797 | 0.031122666 | 0.212944559 |
| M04 - M25 | 2.138173528 | 0.032502662 | 0.220452838 |
| M18 - M37 | -2.13301363 | 0.032923608 | 0.221382882 |
| M03 - M22 | -2.127839521 | 0.033350391 | 0.222335943 |
| M09 - M37 | -2.119986936 | 0.034007147 | 0.224793006 |
| M22 - M26 | 2.103642176 | 0.035409672 | 0.226389706 |
| M10 - M28 | -2.106017373 | 0.035202843 | 0.22692742 |
| M08 - M10 | 2.107952706 | 0.035035081 | 0.227728023 |
| M16 - M37 | -2.109460829 | 0.034904824 | 0.22878792 |
| M10 - M30 | -2.091337162 | 0.036497851 | 0.231449785 |
| M12 - M27 | -2.07169918 | 0.038293504 | 0.235188449 |
| M15 - M27 | -2.07169918 | 0.038293504 | 0.237055024 |
| M23 - M27 | -2.07169918 | 0.038293504 | 0.238951464 |
| M25 - M29 | -2.061857724 | 0.039221283 | 0.239004696 |
| M10 - M13 | -2.074323244 | 0.038049301 | 0.239342374 |
| M19 - M20 | -2.053447615 | 0.040029185 | 0.242036935 |
| M12 - M34 | -2.022819785 | 0.043091731 | 0.247143751 |
| M37 - M39 | 2.041015283 | 0.041249308 | 0.247495848 |
| M26 - M40 | -2.032225641 | 0.042130818 | 0.248954835 |
| M15 - M34 | -2.022819785 | 0.043091731 | 0.248974446 |
| M04 - M32 | 2.028074521 | 0.042552643 | 0.249556853 |
| M19 - M31 | -2.033411249 | 0.042010992 | 0.250141784 |
| M23 - M34 | -2.022819785 | 0.043091731 | 0.250832464 |
| M05 - M22 | -1.998217383 | 0.045693098 | 0.258265335 |
| M12 - M13 | -1.952676574 | 0.050857931 | 0.259275728 |
| M08 - M40 | -1.984320542 | 0.047220118 | 0.259378111 |
| M21 - M27 | -1.987120541 | 0.04690904 | 0.25949682 |
| M12 - M30 | -1.963133947 | 0.049630612 | 0.259811258 |
| M03 - M37 | -1.998695784 | 0.045641279 | 0.25985546 |
| M05 - M40 | -1.947325647 | 0.051495706 | 0.260822406 |
| M07 - M19 | 1.990935344 | 0.046487996 | 0.260867891 |
| M13 - M15 | 1.952676574 | 0.050857931 | 0.260981489 |
| M04 - M24 | 1.987573672 | 0.046858861 | 0.261070794 |
| M15 - M30 | -1.963133947 | 0.049630612 | 0.261566739 |
| M08 - M22 | -1.95599604 | 0.050465621 | 0.262421227 |
| M06 - M12 | 1.967148974 | 0.049166039 | 0.262667878 |
| M13 - M23 | 1.952676574 | 0.050857931 | 0.262709843 |
| M23 - M30 | -1.963133947 | 0.049630612 | 0.263346105 |
| M06 - M15 | 1.967148974 | 0.049166039 | 0.26447938 |
| M21 - M34 | -1.972093187 | 0.04859897 | 0.265085289 |
| M12 - M28 | -1.928397398 | 0.053805715 | 0.265623151 |
| M06 - M23 | 1.967148974 | 0.049166039 | 0.266316043 |
| M04 - M14 | 1.882480503 | 0.059770805 | 0.266407015 |
| M15 - M28 | -1.928397398 | 0.053805715 | 0.267315018 |
| M01 - M22 | -1.878449887 | 0.060319646 | 0.267325706 |
| M26 - M37 | -1.933667518 | 0.05315402 | 0.267484746 |
| M11 - M12 | 1.882834485 | 0.059722802 | 0.267722906 |
| M23 - M28 | -1.928397398 | 0.053805715 | 0.269028576 |
| M11 - M15 | 1.882834485 | 0.059722802 | 0.269270437 |
| M29 - M32 | 1.887792659 | 0.059053791 | 0.26936817 |
| M06 - M10 | 1.889949982 | 0.05876465 | 0.269626041 |
| M08 - M12 | 1.892440369 | 0.058432332 | 0.269687685 |
| M11 - M23 | 1.882834485 | 0.059722802 | 0.270835963 |
| M08 - M15 | 1.892440369 | 0.058432332 | 0.271292968 |
| M10 - M11 | -1.863670775 | 0.062367952 | 0.271770964 |
| M27 - M33 | 1.865848697 | 0.062062537 | 0.271959432 |
| M34 - M36 | 1.907003257 | 0.056520166 | 0.272134131 |
| M13 - M17 | -1.91467407 | 0.055534082 | 0.272431348 |
| M08 - M23 | 1.892440369 | 0.058432332 | 0.272917477 |
| M21 - M30 | -1.866738419 | 0.061938126 | 0.272947673 |
| M24 - M29 | -1.894362375 | 0.058176927 | 0.273361464 |
| M19 - M35 | -1.907646189 | 0.056436961 | 0.273421302 |
| M33 - M34 | -1.910146833 | 0.05611431 | 0.273557263 |
| M17 - M30 | 1.896276113 | 0.057923543 | 0.273820386 |
| M17 - M38 | 1.896538947 | 0.057888815 | 0.275324852 |
| M27 - M36 | 1.898832129 | 0.057586551 | 0.275567546 |
| M13 - M21 | 1.85323801 | 0.063848252 | 0.276675758 |
| M21 - M28 | -1.838608991 | 0.065972718 | 0.28430232 |
| M01 - M40 | -1.833620135 | 0.066710401 | 0.28590172 |
| M05 - M37 | -1.821249672 | 0.068568913 | 0.29226094 |
| M08 - M37 | -1.811683904 | 0.070035052 | 0.29369538 |
| M06 - M21 | 1.81587653 | 0.069389314 | 0.294150354 |
| M07 - M17 | -1.812863466 | 0.069852882 | 0.294514855 |
| M12 - M38 | -1.798009063 | 0.072175572 | 0.294748408 |
| M15 - M38 | -1.798009063 | 0.072175572 | 0.296299715 |
| M08 - M21 | 1.804986877 | 0.071076736 | 0.296469806 |
| M14 - M29 | -1.791744734 | 0.073173865 | 0.297268828 |
| M22 - M28 | 1.786184295 | 0.074069423 | 0.297804897 |
| M23 - M38 | -1.798009063 | 0.072175572 | 0.297867438 |
| M18 - M29 | -1.788408487 | 0.073710128 | 0.297895854 |
| M17 - M35 | 1.798763982 | 0.072056023 | 0.29895584 |
| M20 - M25 | 1.781422734 | 0.074843417 | 0.299373668 |
| M30 - M36 | 1.77155602 | 0.076468286 | 0.304312566 |
| M10 - M38 | -1.768880337 | 0.076913846 | 0.30453198 |
| M28 - M40 | -1.759081212 | 0.078563716 | 0.309493425 |
| M13 - M36 | 1.756247995 | 0.079046073 | 0.309828828 |
| M25 - M31 | -1.745926498 | 0.080823725 | 0.31209161 |
| M28 - M36 | 1.74796694 | 0.080469752 | 0.31227068 |
| M09 - M29 | -1.748571974 | 0.080365034 | 0.313423634 |
| M30 - M33 | 1.736622732 | 0.082453784 | 0.31526447 |
| M11 - M21 | 1.738822878 | 0.082065923 | 0.315327192 |
| M04 - M18 | 1.731760308 | 0.083316248 | 0.317008163 |
| M22 - M34 | 1.729423804 | 0.083733273 | 0.317048314 |
| M16 - M29 | -1.716585901 | 0.08605486 | 0.319632339 |
| M28 - M33 | 1.722540066 | 0.084971731 | 0.320183336 |
| M13 - M33 | 1.719608813 | 0.085503573 | 0.320638399 |
| M08 - M36 | 1.716883044 | 0.086000544 | 0.320958966 |
| M04 - M09 | 1.705199743 | 0.088157178 | 0.325889094 |
| M06 - M36 | 1.697776188 | 0.089550012 | 0.326397239 |
| M34 - M40 | -1.698851296 | 0.089347206 | 0.327186951 |
| M07 - M25 | 1.68852158 | 0.09131115 | 0.328215194 |
| M08 - M33 | 1.699164523 | 0.089288189 | 0.328513147 |
| M04 - M16 | 1.685555991 | 0.091881352 | 0.328749791 |
| M01 - M37 | -1.691582763 | 0.090725555 | 0.329143875 |
| M19 - M27 | -1.689326397 | 0.091156897 | 0.329177685 |
| M26 - M29 | -1.675278069 | 0.093879686 | 0.334366006 |
| M19 - M34 | -1.649158397 | 0.099115189 | 0.349818313 |
| M29 - M39 | 1.650630123 | 0.098814124 | 0.350340985 |
| M21 - M38 | -1.640926884 | 0.100812594 | 0.35420641 |
| M22 - M38 | 1.637822008 | 0.101458829 | 0.354878416 |
| M06 - M33 | 1.631409635 | 0.102803913 | 0.357977912 |
| M11 - M36 | 1.61849649 | 0.105555643 | 0.365926228 |
| M03 - M12 | 1.594662981 | 0.11078762 | 0.366162474 |
| M03 - M15 | 1.594662981 | 0.11078762 | 0.367720612 |
| M22 - M30 | 1.589366373 | 0.111977702 | 0.368534209 |
| M01 - M12 | 1.607776022 | 0.107884242 | 0.369077669 |
| M03 - M23 | 1.594662981 | 0.11078762 | 0.369292068 |
| M13 - M22 | -1.602866782 | 0.108964069 | 0.36953032 |
| M04 - M39 | 1.604240247 | 0.108661106 | 0.370112066 |
| M28 - M37 | -1.595641055 | 0.110568956 | 0.370145002 |
| M01 - M15 | 1.607776022 | 0.107884242 | 0.370703562 |
| M20 - M24 | 1.596113062 | 0.110463553 | 0.371386083 |
| M25 - M35 | -1.597582989 | 0.110135815 | 0.371887167 |
| M01 - M23 | 1.607776022 | 0.107884242 | 0.372343843 |
| M11 - M17 | -1.574979667 | 0.115261138 | 0.377746587 |
| M20 - M32 | 1.572870319 | 0.115748849 | 0.37775775 |
| M03 - M29 | -1.564803136 | 0.117629076 | 0.379135039 |
| M04 - M26 | 1.568649498 | 0.116729632 | 0.379371305 |
| M18 - M20 | -1.565836891 | 0.117386807 | 0.379924107 |
| M38 - M40 | -1.554434358 | 0.120080843 | 0.38386499 |
| M12 - M14 | -1.539034082 | 0.123795976 | 0.384704627 |
| M24 - M31 | -1.548958633 | 0.121391665 | 0.3849004 |
| M11 - M33 | 1.554655955 | 0.12002803 | 0.385275157 |
| M19 - M30 | -1.549579604 | 0.121242451 | 0.385996376 |
| M13 - M19 | 1.535077838 | 0.124764709 | 0.386176481 |
| M14 - M15 | 1.539034082 | 0.123795976 | 0.386243445 |
| M34 - M37 | -1.530290971 | 0.125944722 | 0.386759383 |
| M31 - M32 | 1.531533405 | 0.125637617 | 0.38734127 |
| M06 - M19 | 1.543421572 | 0.122728523 | 0.387563757 |
| M14 - M23 | 1.539034082 | 0.123795976 | 0.387794624 |
| M03 - M04 | -1.53956312 | 0.123666881 | 0.388952287 |
| M13 - M40 | -1.524781341 | 0.127313646 | 0.389429975 |
| M03 - M10 | 1.507874678 | 0.131586605 | 0.393247324 |
| M19 - M28 | -1.508776465 | 0.131355917 | 0.394067751 |
| M12 - M32 | -1.500369642 | 0.133518679 | 0.394487005 |
| M30 - M40 | -1.508807441 | 0.131347999 | 0.395565402 |
| M15 - M32 | -1.500369642 | 0.133518679 | 0.395986956 |
| M09 - M20 | -1.509664265 | 0.131129114 | 0.396436856 |
| M20 - M26 | 1.510579422 | 0.13089564 | 0.397270814 |
| M23 - M32 | -1.500369642 | 0.133518679 | 0.397498357 |
| M36 - M38 | -1.511775854 | 0.130590894 | 0.39789413 |
| M04 - M17 | -1.485009348 | 0.137541348 | 0.400306908 |
| M05 - M29 | -1.485683092 | 0.137362965 | 0.401285066 |
| M18 - M31 | -1.487499648 | 0.136882896 | 0.401385936 |
| M07 - M24 | 1.47997144 | 0.138880869 | 0.402702891 |
| M14 - M20 | -1.487636454 | 0.136846794 | 0.402794338 |
| M01 - M10 | 1.474172578 | 0.140435131 | 0.40570149 |
| M07 - M32 | 1.470121111 | 0.141528956 | 0.407352714 |
| M08 - M19 | 1.463948961 | 0.143207894 | 0.409165412 |
| M16 - M20 | -1.464921363 | 0.142942374 | 0.409908277 |
| M22 - M35 | 1.457101986 | 0.14508823 | 0.413024889 |
| M10 - M14 | -1.45063575 | 0.146881315 | 0.416608821 |
| M17 - M27 | 1.443762335 | 0.14880584 | 0.419020056 |
| M33 - M38 | -1.441849791 | 0.149344753 | 0.419024846 |
| M07 - M22 | -1.445333165 | 0.148364327 | 0.419290488 |
| M11 - M19 | 1.43361065 | 0.151683403 | 0.424061126 |
| M14 - M31 | -1.429256676 | 0.152930473 | 0.424504516 |
| M09 - M31 | -1.430137787 | 0.152677477 | 0.425315828 |
| M26 - M31 | -1.422202607 | 0.154967442 | 0.428633351 |
| M12 - M24 | -1.411166072 | 0.158195652 | 0.429939402 |
| M15 - M24 | -1.411166072 | 0.158195652 | 0.431442686 |
| M11 - M22 | -1.416154715 | 0.156730211 | 0.431977261 |
| M23 - M24 | -1.411166072 | 0.158195652 | 0.43295652 |
| M37 - M38 | 1.412396986 | 0.157833104 | 0.433485284 |
| M01 - M21 | 1.400299006 | 0.161423798 | 0.435676687 |
| M20 - M39 | 1.402034996 | 0.160904797 | 0.435783824 |
| M07 - M18 | 1.391549803 | 0.164058768 | 0.438239176 |
| M10 - M32 | -1.392494434 | 0.163772726 | 0.438978441 |
| M03 - M21 | 1.394101755 | 0.163286879 | 0.4391854 |
| M16 - M31 | -1.384216592 | 0.166292144 | 0.442688985 |
| M25 - M27 | -1.379203469 | 0.167832029 | 0.443759262 |
| M12 - M39 | -1.369677894 | 0.170787472 | 0.444047428 |
| M04 - M05 | 1.376549549 | 0.168651558 | 0.444419647 |
| M24 - M35 | -1.380126938 | 0.167547565 | 0.444513947 |
| M15 - M39 | -1.369677894 | 0.170787472 | 0.445532537 |
| M13 - M37 | -1.372095753 | 0.170033631 | 0.446552971 |
| M35 - M40 | -1.364304267 | 0.172471823 | 0.446936951 |
| M23 - M39 | -1.369677894 | 0.170787472 | 0.447027612 |
| M32 - M35 | -1.359928344 | 0.1738526 | 0.449023273 |
| M07 - M40 | -1.3545283 | 0.175567891 | 0.450470246 |
| M30 - M37 | -1.356121853 | 0.175060401 | 0.450650537 |
| M07 - M14 | 1.348238614 | 0.177581649 | 0.454143233 |
| M08 - M29 | -1.338903234 | 0.180602177 | 0.460358489 |
| M12 - M16 | -1.323040116 | 0.185822038 | 0.463070892 |
| M19 - M38 | -1.320282719 | 0.186740643 | 0.46387803 |
| M15 - M16 | -1.323040116 | 0.185822038 | 0.464555094 |
| M07 - M26 | 1.326025003 | 0.184831416 | 0.465059691 |
| M14 - M21 | 1.327793948 | 0.184246187 | 0.465087462 |
| M07 - M09 | 1.330807395 | 0.183252394 | 0.465592402 |
| M18 - M35 | -1.328661787 | 0.183959577 | 0.465871657 |
| M16 - M23 | 1.323040116 | 0.185822038 | 0.46604884 |
| M31 - M39 | 1.313706899 | 0.188944858 | 0.466382878 |
| M01 - M29 | -1.313908521 | 0.188876991 | 0.467695406 |
| M25 - M34 | -1.305919703 | 0.191579865 | 0.471395252 |
| M07 - M16 | 1.282084543 | 0.19981299 | 0.476618142 |
| M17 - M31 | 1.282453375 | 0.199683651 | 0.477770698 |
| M26 - M35 | -1.283840451 | 0.199197787 | 0.478074688 |
| M21 - M32 | -1.278058751 | 0.201228712 | 0.478531692 |
| M22 - M27 | 1.291628352 | 0.196485881 | 0.478934334 |
| M05 - M12 | 1.284401989 | 0.199001338 | 0.479077294 |
| M03 - M20 | -1.292113131 | 0.196317969 | 0.480025128 |
| M05 - M20 | -1.274539244 | 0.202472374 | 0.480025689 |
| M11 - M40 | -1.293819871 | 0.195727647 | 0.480086682 |
| M04 - M08 | 1.288418422 | 0.197600349 | 0.480150382 |
| M05 - M15 | 1.284401989 | 0.199001338 | 0.480560505 |
| M05 - M23 | 1.284401989 | 0.199001338 | 0.482052929 |
| M09 - M35 | -1.261535292 | 0.207116053 | 0.48806804 |
| M06 - M25 | 1.262975347 | 0.20659804 | 0.488322639 |
| M14 - M35 | -1.24571959 | 0.212867359 | 0.500110061 |
| M01 - M36 | 1.242745708 | 0.213961555 | 0.501171209 |
| M03 - M36 | 1.23587125 | 0.216506425 | 0.505613808 |
| M26 - M27 | -1.210127601 | 0.226229933 | 0.518998083 |
| M16 - M35 | -1.207523716 | 0.227230517 | 0.51976482 |
| M09 - M12 | 1.210188272 | 0.226206657 | 0.520475495 |
| M03 - M31 | -1.196130056 | 0.23164581 | 0.520702398 |
| M07 - M39 | 1.204044615 | 0.228572335 | 0.521305326 |
| M06 - M26 | 1.196535927 | 0.231487487 | 0.521850405 |
| M09 - M15 | 1.210188272 | 0.226206657 | 0.522015363 |
| M10 - M24 | -1.212919569 | 0.225160573 | 0.522694187 |
| M04 - M22 | -1.191897478 | 0.233301447 | 0.522917037 |
| M13 - M25 | 1.197005216 | 0.231304522 | 0.522949354 |
| M18 - M27 | -1.200034514 | 0.230125936 | 0.523318456 |
| M35 - M37 | -1.198149616 | 0.230858776 | 0.523458852 |
| M09 - M23 | 1.210188272 | 0.226206657 | 0.52356437 |
| M01 - M04 | -1.189101975 | 0.234399541 | 0.523872901 |
| M25 - M30 | -1.212979115 | 0.225137805 | 0.524201457 |
| M07 - M37 | -1.183820624 | 0.236484076 | 0.527021656 |
| M01 - M33 | 1.147142032 | 0.251322925 | 0.535606233 |
| M02 - M12 | 1.148762104 | 0.250654089 | 0.535644356 |
| M02 - M15 | 1.148762104 | 0.250654089 | 0.537115906 |
| M12 - M25 | -1.152701523 | 0.249032916 | 0.538076661 |
| M06 - M18 | 1.150667425 | 0.249869083 | 0.538391946 |
| M03 - M33 | 1.142243412 | 0.253352856 | 0.53846111 |
| M02 - M23 | 1.148762104 | 0.250654089 | 0.538595564 |
| M05 - M31 | -1.168399063 | 0.242645833 | 0.539212961 |
| M15 - M25 | -1.152701523 | 0.249032916 | 0.539571318 |
| M22 - M31 | 1.138943625 | 0.254726669 | 0.539909787 |
| M23 - M25 | -1.152701523 | 0.249032916 | 0.541074302 |
| M11 - M37 | -1.158863603 | 0.246511787 | 0.541631532 |
| M21 - M24 | -1.156559852 | 0.247452233 | 0.542170623 |
| M27 - M40 | -1.152767079 | 0.249006 | 0.54252704 |
| M25 - M28 | -1.154417233 | 0.248329153 | 0.542567898 |
| M24 - M27 | -1.158894909 | 0.246499024 | 0.543133443 |
| M14 - M36 | 1.160425281 | 0.245875699 | 0.543294745 |
| M28 - M29 | -1.160464805 | 0.245859615 | 0.544802557 |
| M35 - M39 | 1.131364544 | 0.257901688 | 0.54515804 |
| M09 - M27 | -1.121363212 | 0.262133289 | 0.552605311 |
| M27 - M32 | 1.118275456 | 0.263449366 | 0.553882764 |
| M11 - M25 | 1.111922293 | 0.266171563 | 0.558101664 |
| M10 - M39 | -1.105312543 | 0.269024185 | 0.562570681 |
| M32 - M36 | 1.102969451 | 0.270040432 | 0.563185927 |
| M21 - M39 | -1.099126049 | 0.271713089 | 0.565163225 |
| M26 - M34 | -1.096658168 | 0.27279085 | 0.565895911 |
| M08 - M25 | 1.093115174 | 0.274343234 | 0.567606692 |
| M18 - M34 | -1.083518796 | 0.278578213 | 0.573327192 |
| M01 - M20 | -1.080983545 | 0.279704433 | 0.574130153 |
| M29 - M34 | 1.083758853 | 0.278471733 | 0.574624212 |
| M06 - M09 | 1.072491377 | 0.283499385 | 0.577361672 |
| M12 - M18 | -1.058763801 | 0.289707363 | 0.577932847 |
| M26 - M30 | -1.073306647 | 0.283133554 | 0.578126105 |
| M03 - M07 | -1.074895628 | 0.28242146 | 0.578185666 |
| M15 - M18 | -1.058763801 | 0.289707363 | 0.579414726 |
| M06 - M24 | 1.067430188 | 0.285777623 | 0.580485796 |
| M24 - M34 | -1.05405119 | 0.291859481 | 0.580740804 |
| M18 - M23 | 1.058763801 | 0.289707363 | 0.580904224 |
| M13 - M26 | 1.062849275 | 0.28785031 | 0.581666429 |
| M08 - M20 | -1.051000474 | 0.293258372 | 0.582039518 |
| M01 - M19 | 1.063772539 | 0.287431754 | 0.582329268 |
| M16 - M27 | -1.058763801 | 0.289707363 | 0.5824014 |
| M05 - M07 | -1.046881779 | 0.295154107 | 0.582835957 |
| M14 - M33 | 1.059759485 | 0.289254033 | 0.582992624 |
| M29 - M38 | 1.044878375 | 0.296079186 | 0.583186275 |
| M04 - M40 | -1.047819544 | 0.294721756 | 0.583459314 |
| M11 - M26 | 1.042026719 | 0.297399292 | 0.584310951 |
| M16 - M21 | 1.040133408 | 0.298277924 | 0.584564776 |
| M03 - M19 | 1.036277606 | 0.300072651 | 0.585141669 |
| M18 - M30 | -1.0370769 | 0.29970002 | 0.585879738 |
| M14 - M27 | -1.032157201 | 0.301998481 | 0.585967202 |
| M10 - M16 | -1.033061609 | 0.301575068 | 0.586604871 |
| M04 - M28 | 1.022047693 | 0.306758328 | 0.590793817 |
| M13 - M18 | 1.02357649 | 0.306035354 | 0.590860337 |
| M06 - M32 | 1.024399121 | 0.305646797 | 0.591574446 |
| M27 - M37 | -1.010729707 | 0.312145815 | 0.595290307 |
| M32 - M34 | -1.011720352 | 0.311671782 | 0.595843112 |
| M06 - M16 | 1.011863271 | 0.311603433 | 0.597176112 |
| M26 - M28 | -1.013261907 | 0.310935077 | 0.597362956 |
| M02 - M22 | -1.000271305 | 0.31717923 | 0.603414145 |
| M03 - M35 | -0.991962149 | 0.32121599 | 0.603731259 |
| M05 - M35 | -0.990204506 | 0.32207417 | 0.60388907 |
| M05 - M21 | 0.994857165 | 0.319805739 | 0.603991468 |
| M27 - M39 | 0.992808024 | 0.320803519 | 0.604412427 |
| M32 - M33 | 0.995011342 | 0.319730748 | 0.605315494 |
| M09 - M34 | -0.995612147 | 0.319438631 | 0.606233898 |
| M11 - M18 | 0.981448996 | 0.326371375 | 0.610478831 |
| M31 - M40 | -0.975860058 | 0.329133818 | 0.611248519 |
| M13 - M29 | -0.976930343 | 0.328603639 | 0.611720378 |
| M25 - M38 | -0.97753619 | 0.32830377 | 0.61262426 |
| M08 - M26 | 0.957934784 | 0.338095645 | 0.614719354 |
| M06 - M17 | -0.949783583 | 0.342222229 | 0.615053776 |
| M14 - M19 | 0.952222878 | 0.340983961 | 0.615665484 |
| M01 - M31 | -0.953736365 | 0.340217108 | 0.615706135 |
| M06 - M22 | -0.955172047 | 0.339490699 | 0.615820338 |
| M24 - M30 | -0.967465365 | 0.333311447 | 0.616073291 |
| M06 - M39 | 0.958010604 | 0.338057412 | 0.616085937 |
| M05 - M10 | 0.959417715 | 0.337348354 | 0.616233528 |
| M13 - M24 | 0.950036412 | 0.342093751 | 0.616242785 |
| M02 - M17 | -0.960888366 | 0.336608301 | 0.616325058 |
| M18 - M28 | -0.968452648 | 0.332818357 | 0.616623083 |
| M09 - M30 | -0.946306143 | 0.343992461 | 0.616814069 |
| M06 - M14 | 0.960959074 | 0.336572746 | 0.617709981 |
| M24 - M36 | 0.96406395 | 0.335013865 | 0.617756063 |
| M29 - M30 | 0.961622317 | 0.336239357 | 0.618553534 |
| M26 - M38 | -0.936437485 | 0.349047986 | 0.624443644 |
| M09 - M13 | -0.931804377 | 0.351437632 | 0.627279985 |
| M16 - M34 | -0.924295272 | 0.355332604 | 0.631342667 |
| M04 - M34 | 0.925577828 | 0.35466542 | 0.631595954 |
| M08 - M31 | -0.921071404 | 0.357013149 | 0.632886945 |
| M30 - M32 | 0.917710667 | 0.358770362 | 0.634559824 |
| M05 - M06 | -0.89481777 | 0.37088451 | 0.63860909 |
| M20 - M28 | 0.899104299 | 0.368597109 | 0.638901655 |
| M09 - M11 | -0.8898273 | 0.373558625 | 0.638981859 |
| M02 - M21 | 0.892715251 | 0.372009681 | 0.639135576 |
| M05 - M27 | -0.897309119 | 0.369553992 | 0.639139941 |
| M24 - M28 | -0.890955169 | 0.372953221 | 0.639348379 |
| M12 - M33 | -0.905864287 | 0.365007689 | 0.639788758 |
| M19 - M32 | -0.89506657 | 0.370751503 | 0.639792417 |
| M13 - M32 | 0.89931271 | 0.368486119 | 0.640131789 |
| M14 - M34 | -0.901740805 | 0.367194571 | 0.640742205 |
| M09 - M21 | 0.899885654 | 0.368181105 | 0.641029603 |
| M15 - M33 | -0.905864287 | 0.365007689 | 0.641229724 |
| M08 - M18 | 0.902137285 | 0.366983945 | 0.641810486 |
| M23 - M33 | -0.905864287 | 0.365007689 | 0.642677195 |
| M36 - M39 | -0.906024106 | 0.364923094 | 0.64398193 |
| M11 - M24 | 0.880386985 | 0.378649705 | 0.646273019 |
| M16 - M30 | -0.873485202 | 0.382398669 | 0.651246641 |
| M09 - M28 | -0.871133966 | 0.38368101 | 0.652006945 |
| M04 - M37 | -0.869093104 | 0.384796208 | 0.652480527 |
| M02 - M40 | -0.866133068 | 0.386417193 | 0.653807831 |
| M04 - M38 | 0.857419334 | 0.391213178 | 0.659063237 |
| M18 - M38 | -0.853840858 | 0.393193173 | 0.659549838 |
| M13 - M16 | 0.858177177 | 0.390794637 | 0.659783153 |
| M09 - M10 | 0.854701456 | 0.392716445 | 0.660169885 |
| M34 - M39 | 0.847140513 | 0.396916806 | 0.664367186 |
| M03 - M27 | -0.844195131 | 0.398560375 | 0.665689706 |
| M16 - M36 | 0.840068328 | 0.400870077 | 0.668116795 |
| M28 - M32 | 0.836662693 | 0.402782195 | 0.668447047 |
| M24 - M33 | 0.837789805 | 0.402148765 | 0.668818842 |
| M21 - M25 | -0.822234615 | 0.410943382 | 0.669177114 |
| M11 - M16 | 0.817522767 | 0.413629744 | 0.669359337 |
| M20 - M34 | 0.818778492 | 0.412912804 | 0.669588331 |
| M31 - M37 | -0.823174471 | 0.410408784 | 0.669704711 |
| M03 - M06 | -0.823721572 | 0.410097779 | 0.670600142 |
| M11 - M29 | -0.819177671 | 0.412685052 | 0.67061321 |
| M20 - M38 | 0.824473365 | 0.409670644 | 0.671309038 |
| M20 - M22 | -0.83176682 | 0.405540576 | 0.671595858 |
| M17 - M20 | 0.830028347 | 0.406522757 | 0.671796081 |
| M14 - M30 | -0.824951146 | 0.409399327 | 0.67227679 |
| M02 - M10 | 0.826449287 | 0.408549275 | 0.672296275 |
| M11 - M32 | 0.827918999 | 0.407716374 | 0.672344127 |
| M08 - M24 | 0.811988257 | 0.41679837 | 0.673090535 |
| M08 - M09 | 0.797953092 | 0.424897712 | 0.674990255 |
| M12 - M19 | -0.799126697 | 0.424216951 | 0.675284126 |
| M01 - M07 | -0.805630242 | 0.420456083 | 0.676197412 |
| M13 - M14 | 0.806838446 | 0.419759566 | 0.676472028 |
| M15 - M19 | -0.799126697 | 0.424216951 | 0.676665075 |
| M30 - M39 | 0.802975509 | 0.42198889 | 0.67726612 |
| M02 - M37 | -0.792214476 | 0.428235629 | 0.677533044 |
| M05 - M36 | 0.801057968 | 0.423098088 | 0.677651968 |
| M19 - M23 | 0.799126697 | 0.424216951 | 0.678051684 |
| M16 - M28 | -0.79231116 | 0.428179266 | 0.678820788 |
| M13 - M39 | 0.787667484 | 0.43089123 | 0.680354573 |
| M33 - M39 | -0.778281997 | 0.436402786 | 0.686278575 |
| M04 - M13 | 0.773175323 | 0.439418629 | 0.686866794 |
| M29 - M35 | 0.774618948 | 0.438564857 | 0.686908813 |
| M06 - M40 | -0.77836457 | 0.43635412 | 0.68758831 |
| M10 - M25 | -0.770768603 | 0.4408441 | 0.687716796 |
| M19 - M24 | -0.775004004 | 0.438337294 | 0.687933781 |
| M05 - M34 | -0.741759414 | 0.458233115 | 0.695373209 |
| M01 - M35 | -0.742551928 | 0.457753002 | 0.695998716 |
| M11 - M14 | 0.751160396 | 0.452556131 | 0.696240202 |
| M07 - M29 | -0.749007286 | 0.453852814 | 0.69686062 |
| M19 - M39 | -0.742721088 | 0.457650559 | 0.697202024 |
| M02 - M36 | 0.751484879 | 0.452360898 | 0.697315218 |
| M01 - M26 | 0.746204444 | 0.455543924 | 0.698083027 |
| M08 - M32 | 0.752020216 | 0.452038902 | 0.6981987 |
| M13 - M20 | -0.743017751 | 0.457470933 | 0.698292227 |
| M07 - M08 | 0.756901873 | 0.449108653 | 0.699211077 |
| M28 - M31 | -0.743444925 | 0.457212354 | 0.699265953 |
| M09 - M38 | -0.752319534 | 0.451858923 | 0.699305477 |
| M04 - M30 | 0.753559683 | 0.451113661 | 0.699540071 |
| M11 - M39 | 0.7546364 | 0.450467177 | 0.69992908 |
| M14 - M28 | -0.735258401 | 0.462182137 | 0.700004014 |
| M20 - M30 | 0.728515985 | 0.466297787 | 0.700794362 |
| M24 - M38 | -0.726152838 | 0.46774508 | 0.70161762 |
| M12 - M36 | -0.729022105 | 0.465988141 | 0.701680985 |
| M15 - M36 | -0.729022105 | 0.465988141 | 0.703038201 |
| M23 - M36 | -0.729022105 | 0.465988141 | 0.704400679 |
| M28 - M39 | 0.717280334 | 0.473201139 | 0.705730189 |
| M05 - M30 | -0.717976315 | 0.472771891 | 0.706440756 |
| M18 - M21 | 0.718507953 | 0.472444146 | 0.707306015 |
| M08 - M16 | 0.712809345 | 0.475963742 | 0.708495647 |
| M22 - M29 | 0.703254379 | 0.481897236 | 0.709207253 |
| M12 - M26 | -0.703470248 | 0.481762742 | 0.7103496 |
| M15 - M26 | -0.703470248 | 0.481762742 | 0.71169496 |
| M01 - M25 | 0.698350413 | 0.484958079 | 0.712367799 |
| M23 - M26 | -0.703470248 | 0.481762742 | 0.713045424 |
| M16 - M33 | 0.706031063 | 0.48016882 | 0.713393676 |
| M05 - M13 | -0.704475906 | 0.48113645 | 0.713472302 |
| M03 - M26 | 0.694780817 | 0.487192672 | 0.714305045 |
| M10 - M12 | 0.685380338 | 0.493103948 | 0.716240371 |
| M09 - M36 | 0.689102186 | 0.490758964 | 0.716838937 |
| M01 - M06 | -0.683078566 | 0.494557198 | 0.717016012 |
| M05 - M11 | -0.690151348 | 0.490099015 | 0.71721807 |
| M10 - M15 | 0.685380338 | 0.493103948 | 0.717576641 |
| M16 - M19 | 0.679834722 | 0.496609119 | 0.718655125 |
| M10 - M23 | 0.685380338 | 0.493103948 | 0.718917905 |
| M16 - M38 | -0.671707526 | 0.501769909 | 0.720774455 |
| M02 - M33 | 0.662934014 | 0.507372816 | 0.720857553 |
| M05 - M19 | 0.661469734 | 0.508311118 | 0.720877586 |
| M08 - M35 | -0.669876964 | 0.502936226 | 0.721121795 |
| M03 - M34 | -0.668397328 | 0.50388 | 0.721149358 |
| M32 - M38 | -0.665498843 | 0.505731483 | 0.721152755 |
| M05 - M33 | 0.672592952 | 0.501206287 | 0.72129318 |
| M02 - M19 | 0.674011981 | 0.500303697 | 0.721325108 |
| M06 - M37 | -0.66385038 | 0.506786078 | 0.721337848 |
| M31 - M38 | 0.666131041 | 0.505327345 | 0.721896208 |
| M17 - M29 | 0.674245132 | 0.500155481 | 0.722446806 |
| M27 - M29 | -0.6578221 | 0.510652452 | 0.722883689 |
| M01 - M27 | -0.656086421 | 0.511768521 | 0.723151171 |
| M31 - M34 | 0.648431482 | 0.516705914 | 0.728807618 |
| M08 - M14 | 0.643226779 | 0.520076959 | 0.732238318 |
| M03 - M25 | 0.639719003 | 0.522355299 | 0.73412096 |
| M05 - M28 | -0.633777132 | 0.526226279 | 0.735585121 |
| M08 - M39 | 0.633977112 | 0.526095759 | 0.736722966 |
| M10 - M18 | -0.634238478 | 0.5259252 | 0.737808734 |
| M03 - M30 | -0.628483853 | 0.529687021 | 0.739098169 |
| M20 - M40 | -0.62431943 | 0.532417826 | 0.741581972 |
| M11 - M20 | -0.619836965 | 0.535365129 | 0.744357934 |
| M14 - M26 | 0.617975322 | 0.536591606 | 0.744735681 |
| M02 - M29 | -0.61510429 | 0.538485849 | 0.746037233 |
| M01 - M18 | 0.613212979 | 0.539735524 | 0.746442745 |
| M03 - M13 | -0.611469934 | 0.540888517 | 0.746713351 |
| M38 - M39 | 0.605751749 | 0.544679615 | 0.750618551 |
| M25 - M36 | 0.591307756 | 0.554314234 | 0.762548681 |
| M14 - M38 | -0.585223112 | 0.558397706 | 0.762784958 |
| M03 - M11 | -0.589284075 | 0.555670726 | 0.763068954 |
| M04 - M11 | 0.585524945 | 0.558194799 | 0.763845514 |
| M06 - M08 | 0.586880399 | 0.557284035 | 0.763939451 |
| M26 - M32 | -0.574208025 | 0.56582704 | 0.770235761 |
| M02 - M26 | 0.574381052 | 0.565709972 | 0.771422689 |
| M13 - M31 | -0.568187508 | 0.569907653 | 0.774438971 |
| M05 - M38 | -0.551115463 | 0.581554526 | 0.783441331 |
| M07 - M28 | 0.554662919 | 0.579125244 | 0.784232101 |
| M30 - M31 | -0.551652951 | 0.581186151 | 0.78429965 |
| M03 - M18 | 0.548635738 | 0.583255455 | 0.784378025 |
| M08 - M27 | -0.552655441 | 0.580499371 | 0.784730519 |
| M05 - M08 | -0.554887442 | 0.578971652 | 0.785387633 |
| M09 - M33 | 0.545686636 | 0.58528136 | 0.785747781 |
| M09 - M19 | 0.54378335 | 0.586590568 | 0.786152307 |
| M20 - M35 | 0.532213143 | 0.594578381 | 0.795490802 |
| M14 - M25 | 0.528263428 | 0.597316511 | 0.797785751 |
| M03 - M28 | -0.524545069 | 0.599899482 | 0.799865976 |
| M02 - M20 | -0.511546552 | 0.6089684 | 0.810572273 |
| M26 - M39 | -0.508106315 | 0.611378787 | 0.812394299 |
| M07 - M20 | -0.501855514 | 0.61576916 | 0.814067703 |
| M24 - M26 | 0.502989887 | 0.614971382 | 0.814393342 |
| M21 - M33 | -0.498052302 | 0.618447179 | 0.814845945 |
| M04 - M35 | 0.50326781 | 0.614775995 | 0.815519177 |
| M01 - M09 | 0.495809354 | 0.620028925 | 0.81555238 |
| M18 - M36 | 0.498332573 | 0.618249653 | 0.815964009 |
| M20 - M37 | -0.489363162 | 0.624584611 | 0.820161611 |
| M07 - M38 | 0.48730948 | 0.626039031 | 0.820689822 |
| M28 - M35 | -0.483079392 | 0.629039366 | 0.823239439 |
| M06 - M28 | 0.481135422 | 0.630420254 | 0.823664654 |
| M12 - M21 | -0.472104515 | 0.63685219 | 0.826530297 |
| M29 - M40 | -0.470112824 | 0.638274412 | 0.827000069 |
| M15 - M21 | -0.472104515 | 0.63685219 | 0.827907847 |
| M21 - M23 | 0.472104515 | 0.63685219 | 0.829289997 |
| M06 - M38 | 0.463416372 | 0.643065946 | 0.8304494 |
| M04 - M07 | 0.46487964 | 0.642017652 | 0.830470594 |
| M05 - M26 | 0.472104515 | 0.63685219 | 0.83067677 |
| M16 - M26 | 0.461468538 | 0.644462489 | 0.830877259 |
| M25 - M32 | -0.459453727 | 0.645908374 | 0.831367214 |
| M29 - M31 | 0.450890927 | 0.652068163 | 0.832427442 |
| M20 - M27 | 0.455429687 | 0.648800142 | 0.833713526 |
| M02 - M04 | -0.450936559 | 0.652035273 | 0.833750021 |
| M14 - M18 | 0.447805018 | 0.654293922 | 0.833904018 |
| M01 - M34 | -0.453245164 | 0.650372214 | 0.834359091 |
| M02 - M25 | 0.451546182 | 0.651595948 | 0.834556387 |
| M11 - M31 | -0.442963031 | 0.657792475 | 0.835632134 |
| M01 - M30 | -0.443004975 | 0.657762136 | 0.836956715 |
| M07 - M34 | 0.439835534 | 0.660056229 | 0.837144486 |
| M02 - M18 | 0.438290921 | 0.661175406 | 0.837202625 |
| M04 - M29 | -0.430793242 | 0.666618714 | 0.837298867 |
| M01 - M13 | -0.42769695 | 0.668871771 | 0.837431752 |
| M19 - M25 | -0.436554188 | 0.662434693 | 0.837437699 |
| M35 - M38 | 0.431902674 | 0.665812152 | 0.837634642 |
| M25 - M33 | 0.428751872 | 0.668103807 | 0.837815063 |
| M22 - M37 | 0.433043564 | 0.664983122 | 0.83794319 |
| M01 - M24 | 0.425367991 | 0.670568436 | 0.838210545 |
| M01 - M11 | -0.433584949 | 0.664589866 | 0.838802744 |
| M03 - M09 | 0.419685244 | 0.674715406 | 0.839358879 |
| M03 - M08 | -0.422097565 | 0.672953805 | 0.839846349 |
| M03 - M38 | -0.420196225 | 0.674342112 | 0.840234581 |
| M27 - M28 | 0.412131043 | 0.680243377 | 0.84488827 |
| M06 - M34 | 0.404849465 | 0.685588167 | 0.850172925 |
| M01 - M16 | 0.40267738 | 0.687185576 | 0.85080119 |
| M02 - M31 | -0.396809126 | 0.691508221 | 0.854796216 |
| M27 - M38 | 0.387056275 | 0.698714547 | 0.858263538 |
| M18 - M32 | -0.38948436 | 0.696917878 | 0.858761366 |
| M19 - M21 | 0.387201796 | 0.698606821 | 0.859484732 |
| M06 - M29 | -0.389723248 | 0.696741203 | 0.859902118 |
| M04 - M27 | 0.38022431 | 0.703778915 | 0.863125085 |
| M34 - M35 | -0.368648894 | 0.712389446 | 0.868224638 |
| M10 - M33 | -0.365631266 | 0.714640241 | 0.868254499 |
| M09 - M26 | 0.369380506 | 0.711844126 | 0.868917713 |
| M06 - M13 | 0.372268158 | 0.709693204 | 0.869012087 |
| M07 - M13 | 0.365802327 | 0.714512583 | 0.86945369 |
| M18 - M19 | 0.370172087 | 0.711254273 | 0.869558516 |
| M06 - M30 | 0.360056578 | 0.718804824 | 0.870602116 |
| M02 - M09 | 0.360058517 | 0.718803373 | 0.871954325 |
| M07 - M30 | 0.347148831 | 0.72847951 | 0.876873484 |
| M01 - M32 | 0.350788642 | 0.725746919 | 0.877647437 |
| M25 - M39 | -0.348917893 | 0.727150952 | 0.877984122 |
| M18 - M33 | 0.347413715 | 0.728280531 | 0.877988894 |
| M24 - M25 | 0.342665125 | 0.731850403 | 0.879573673 |
| M21 - M26 | -0.340192959 | 0.73371122 | 0.880453464 |
| M03 - M24 | 0.334127208 | 0.73828357 | 0.880521689 |
| M01 - M39 | 0.336721602 | 0.736326777 | 0.880881727 |
| M01 - M28 | -0.334228224 | 0.738207348 | 0.881779068 |
| M02 - M06 | -0.336721602 | 0.736326777 | 0.882234848 |
| M29 - M37 | -0.328075452 | 0.742854614 | 0.884620762 |
| M17 - M37 | 0.320490115 | 0.748596823 | 0.890099881 |
| M27 - M34 | 0.314241651 | 0.753337511 | 0.891658966 |
| M09 - M14 | -0.306111981 | 0.759519386 | 0.892206508 |
| M18 - M39 | -0.312040022 | 0.755010107 | 0.892284672 |
| M31 - M35 | 0.315051203 | 0.752722778 | 0.89228536 |
| M01 - M05 | 0.310487585 | 0.756190199 | 0.892327315 |
| M22 - M40 | 0.30696759 | 0.758868042 | 0.892785931 |
| M18 - M24 | -0.297778591 | 0.765872156 | 0.892945114 |
| M02 - M24 | 0.299249913 | 0.764749368 | 0.892970819 |
| M21 - M36 | -0.288753454 | 0.772770054 | 0.892978729 |
| M03 - M16 | 0.315622504 | 0.752289055 | 0.893128558 |
| M06 - M11 | 0.30187833 | 0.762744816 | 0.893304739 |
| M05 - M25 | 0.300115634 | 0.764088954 | 0.893537308 |
| M13 - M35 | -0.307367027 | 0.758564023 | 0.893776341 |
| M08 - M34 | -0.29520984 | 0.767833585 | 0.893895816 |
| M02 - M16 | 0.30244947 | 0.762309448 | 0.894137398 |
| M02 - M07 | -0.288774915 | 0.77275363 | 0.894284616 |
| M08 - M30 | -0.291039337 | 0.771021235 | 0.894935362 |
| M30 - M35 | -0.289254327 | 0.772386761 | 0.895188222 |
| M08 - M11 | -0.291858329 | 0.770394945 | 0.895541069 |
| M13 - M27 | -0.273688937 | 0.78432368 | 0.898344303 |
| M08 - M13 | -0.272155542 | 0.785502425 | 0.898375207 |
| M01 - M14 | 0.274573857 | 0.783643653 | 0.898885367 |
| M16 - M25 | 0.280472347 | 0.779115138 | 0.898979005 |
| M02 - M27 | -0.263483587 | 0.792177872 | 0.899415925 |
| M02 - M35 | -0.266208982 | 0.790078266 | 0.899651164 |
| M07 - M31 | -0.275199631 | 0.78316287 | 0.899656905 |
| M01 - M38 | -0.269030146 | 0.787906484 | 0.899805355 |
| M02 - M39 | 0.267194624 | 0.789319315 | 0.900100974 |
| M02 - M05 | 0.263512333 | 0.792155719 | 0.900701838 |
| M25 - M26 | 0.275319973 | 0.783070421 | 0.900877475 |
| M05 - M18 | 0.276349213 | 0.782279861 | 0.901297329 |
| M02 - M32 | 0.257603653 | 0.79671281 | 0.901939031 |
| M27 - M30 | 0.258380912 | 0.796112947 | 0.90256991 |
| M27 - M31 | -0.252350478 | 0.800770175 | 0.903908446 |
| M07 - M11 | 0.250598023 | 0.802124911 | 0.904129234 |
| M16 - M18 | 0.253047381 | 0.800231599 | 0.904609634 |
| M18 - M26 | 0.246466328 | 0.805321267 | 0.905116122 |
| M03 - M39 | 0.243371569 | 0.807717566 | 0.905200721 |
| M20 - M31 | 0.244684341 | 0.806700854 | 0.905362109 |
| M09 - M32 | -0.241520319 | 0.80915187 | 0.905507114 |
| M03 - M32 | 0.246738163 | 0.80511087 | 0.906185395 |
| M10 - M19 | -0.238109094 | 0.811796482 | 0.907165123 |
| M06 - M20 | -0.234916228 | 0.814273754 | 0.908631657 |
| M01 - M08 | -0.23151782 | 0.816912543 | 0.910273977 |
| M02 - M14 | 0.222402441 | 0.824000612 | 0.915556236 |
| M03 - M05 | 0.223456501 | 0.823180234 | 0.915949476 |
| M10 - M21 | 0.211227539 | 0.832709723 | 0.919990912 |
| M33 - M36 | 0.214217683 | 0.830377307 | 0.920020312 |
| M17 - M22 | -0.211931261 | 0.832160662 | 0.920688391 |
| M10 - M26 | -0.214501827 | 0.830155743 | 0.921083185 |
| M11 - M35 | -0.204262915 | 0.838148049 | 0.924689502 |
| M11 - M27 | -0.191968909 | 0.84776656 | 0.931349178 |
| M14 - M16 | 0.19039793 | 0.848997319 | 0.931389464 |
| M04 - M20 | -0.194419466 | 0.845847449 | 0.931865834 |
| M14 - M24 | 0.192379912 | 0.847444626 | 0.932308616 |
| M20 - M29 | -0.171779075 | 0.863611216 | 0.940805515 |
| M11 - M38 | 0.177074769 | 0.85944966 | 0.941531931 |
| M11 - M28 | 0.172281031 | 0.863216596 | 0.941690832 |
| M09 - M39 | -0.174757903 | 0.861269868 | 0.94220266 |
| M06 - M35 | 0.173096828 | 0.862575318 | 0.942309171 |
| M08 - M28 | -0.14559096 | 0.884244305 | 0.947404612 |
| M09 - M24 | -0.147097077 | 0.883055397 | 0.9474322 |
| M13 - M38 | 0.140091626 | 0.888587597 | 0.948150924 |
| M34 - M38 | 0.138534962 | 0.88981764 | 0.948166338 |
| M02 - M30 | -0.136757431 | 0.891222533 | 0.94836777 |
| M03 - M14 | 0.161350086 | 0.871817677 | 0.948420904 |
| M06 - M07 | 0.141309191 | 0.887625689 | 0.948421969 |
| M06 - M27 | 0.147386247 | 0.882827161 | 0.948491991 |
| M28 - M34 | -0.131985798 | 0.894995525 | 0.948500693 |
| M09 - M18 | 0.142511772 | 0.886675782 | 0.948706598 |
| M37 - M40 | -0.147837184 | 0.882471263 | 0.949417359 |
| M13 - M28 | 0.132043818 | 0.894949634 | 0.949742468 |
| M02 - M13 | -0.128962966 | 0.897386953 | 0.949744672 |
| M05 - M09 | 0.148785875 | 0.881722597 | 0.949922134 |
| M28 - M30 | -0.149835914 | 0.880894074 | 0.950342155 |
| M26 - M36 | 0.132549474 | 0.894549694 | 0.950611391 |
| M02 - M34 | -0.124790697 | 0.900689253 | 0.950659834 |
| M09 - M25 | 0.15079896 | 0.880134307 | 0.950837617 |
| M17 - M40 | 0.151929791 | 0.879242311 | 0.951191405 |
| M01 - M03 | 0.125488465 | 0.900136856 | 0.951364157 |
| M04 - M06 | 0.118852725 | 0.905392036 | 0.951759822 |
| M16 - M32 | -0.120261467 | 0.904276028 | 0.951869503 |
| M30 - M38 | 0.155399651 | 0.876506259 | 0.952193429 |
| M02 - M11 | -0.153683514 | 0.877859285 | 0.952336915 |
| M02 - M03 | 0.152107231 | 0.879102361 | 0.952360891 |
| M19 - M36 | 0.121139001 | 0.903580938 | 0.952423151 |
| M05 - M14 | -0.109347737 | 0.912926685 | 0.95325678 |
| M24 - M32 | -0.110617098 | 0.911919989 | 0.953482026 |
| M09 - M16 | -0.111871537 | 0.910925265 | 0.953720412 |
| M10 - M36 | -0.112812863 | 0.91017892 | 0.954219836 |
| M14 - M39 | 0.114238758 | 0.909048528 | 0.954317432 |
| M04 - M31 | 0.099110602 | 0.921050454 | 0.960453682 |
| M14 - M32 | 0.089538645 | 0.928653843 | 0.965799996 |
| M01 - M02 | -0.087209356 | 0.930505102 | 0.966436724 |
| M08 - M38 | -0.090034708 | 0.928259634 | 0.966678925 |
| M19 - M33 | -0.070905726 | 0.943472786 | 0.97600633 |
| M11 - M34 | 0.073827172 | 0.941147906 | 0.976190647 |
| M02 - M28 | -0.072219087 | 0.942427556 | 0.976219779 |
| M16 - M39 | -0.065955778 | 0.947413033 | 0.978784325 |
| M11 - M13 | 0.055370379 | 0.955843394 | 0.98358555 |
| M07 - M35 | 0.055494302 | 0.955744669 | 0.984783147 |
| M06 - M31 | -0.047366127 | 0.962221425 | 0.984951065 |
| M02 - M38 | -0.056593306 | 0.954869167 | 0.985182474 |
| M05 - M32 | -0.047724979 | 0.961935427 | 0.985952211 |
| M24 - M39 | -0.048901795 | 0.960997559 | 0.986286968 |
| M11 - M30 | 0.040868613 | 0.96740064 | 0.98636928 |
| M05 - M16 | 0.049678014 | 0.960378977 | 0.986950728 |
| M19 - M26 | -0.041681069 | 0.966752946 | 0.986999081 |
| M32 - M39 | 0.043106708 | 0.965616472 | 0.987130863 |
| M30 - M34 | 0.034608744 | 0.972391729 | 0.990163902 |
| M05 - M24 | 0.032521894 | 0.974055856 | 0.990565278 |
| M28 - M38 | 0.028181435 | 0.977517444 | 0.991500139 |
| M07 - M27 | 0.029338319 | 0.976594766 | 0.991854059 |
| M16 - M24 | -0.024839007 | 0.980183378 | 0.992913032 |
| M27 - M35 | 0.015031416 | 0.988007117 | 0.995666087 |
| M13 - M34 | 0.016210787 | 0.98706623 | 0.996004734 |
| M13 - M30 | -0.016534557 | 0.986807933 | 0.997033922 |
| M18 - M25 | -0.011648324 | 0.990706193 | 0.997097845 |
| M05 - M39 | -0.009314628 | 0.99256811 | 0.99768444 |
| M02 - M08 | -0.017076817 | 0.986375333 | 0.997889442 |
| M26 - M33 | -0.005982123 | 0.995226985 | 0.99906956 |
| M12 - M15 | 0 | 1 | 1 |
| M12 - M23 | 0 | 1 | 1 |
| M15 - M23 | 0 | 1 | 1 |

**Table S5 Results of a Dunn test with fdr correction for multiple testing for developmental time of metamorphs between matrilines. Given are the respective comparisons of matriline number (M1-M40), the z-value, the unadjusted p-value (res.P.unadj) and the adjusted p-value with fdr correction (res.P.adj). Comparisons are sorted from smallest to largest adjusted p-value.**

| **res.Comparison** | **res.Z** | **res.P.unadj** | **res.P.adj** |
| --- | --- | --- | --- |
| M07 - M24 | -5.712408477 | 1.11E-08 | 8.69E-06 |
| M24 - M32 | 5.462582358 | 4.69E-08 | 1.83E-05 |
| M07 - M40 | -5.24734994 | 1.54E-07 | 4.01E-05 |
| M04 - M24 | -5.1584234 | 2.49E-07 | 4.86E-05 |
| M32 - M40 | -5.03740714 | 4.72E-07 | 5.26E-05 |
| M11 - M24 | -5.064550773 | 4.09E-07 | 5.32E-05 |
| M07 - M31 | -5.06765951 | 4.03E-07 | 6.28E-05 |
| M16 - M24 | -4.843023121 | 1.28E-06 | 8.31E-05 |
| M11 - M40 | -4.846565054 | 1.26E-06 | 8.91E-05 |
| M08 - M24 | -4.906560465 | 9.27E-07 | 9.04E-05 |
| M24 - M29 | 4.795542503 | 1.62E-06 | 9.04E-05 |
| M24 - M33 | 4.878145351 | 1.07E-06 | 9.28E-05 |
| M10 - M24 | -4.802724784 | 1.57E-06 | 9.39E-05 |
| M31 - M32 | 4.848070774 | 1.25E-06 | 9.72E-05 |
| M04 - M40 | -4.702541696 | 2.57E-06 | 0.00013361 |
| M07 - M39 | -4.676293529 | 2.92E-06 | 0.000142402 |
| M11 - M31 | -4.65683939 | 3.21E-06 | 0.000147329 |
| M16 - M40 | -4.610086935 | 4.03E-06 | 0.000174417 |
| M29 - M40 | -4.566015854 | 4.97E-06 | 0.000193862 |
| M33 - M40 | -4.574323424 | 4.78E-06 | 0.000196134 |
| M07 - M25 | -4.484627332 | 7.30E-06 | 0.000227889 |
| M07 - M19 | -4.467474258 | 7.91E-06 | 0.000228651 |
| M04 - M31 | -4.498931175 | 6.83E-06 | 0.000231612 |
| M08 - M40 | -4.51565186 | 6.31E-06 | 0.000234454 |
| M10 - M40 | -4.505560297 | 6.62E-06 | 0.000234702 |
| M32 - M39 | -4.486532098 | 7.24E-06 | 0.000235273 |
| M07 - M38 | -4.469268622 | 7.85E-06 | 0.000235463 |
| M11 - M39 | -4.443387073 | 8.86E-06 | 0.000246685 |
| M16 - M31 | -4.410171078 | 1.03E-05 | 0.000277812 |
| M05 - M24 | -4.361617273 | 1.29E-05 | 0.000314692 |
| M29 - M31 | -4.364770923 | 1.27E-05 | 0.000320193 |
| M31 - M33 | 4.367433715 | 1.26E-05 | 0.000326859 |
| M11 - M19 | -4.326482344 | 1.52E-05 | 0.000358113 |
| M07 - M35 | -4.273243206 | 1.93E-05 | 0.000375668 |
| M19 - M32 | 4.29470385 | 1.75E-05 | 0.000379008 |
| M11 - M38 | -4.276561649 | 1.90E-05 | 0.000379604 |
| M32 - M38 | -4.281855375 | 1.85E-05 | 0.000380438 |
| M08 - M31 | -4.303206524 | 1.68E-05 | 0.0003862 |
| M25 - M32 | 4.284278909 | 1.83E-05 | 0.000386485 |
| M10 - M31 | -4.29611769 | 1.74E-05 | 0.00038736 |
| M11 - M25 | -4.243579888 | 2.20E-05 | 0.000418502 |
| M05 - M40 | -4.218774392 | 2.46E-05 | 0.000456177 |
| M16 - M39 | -4.187340514 | 2.82E-05 | 0.000511974 |
| M07 - M18 | -4.143210123 | 3.42E-05 | 0.000593628 |
| M29 - M39 | -4.144872558 | 3.40E-05 | 0.000602732 |
| M04 - M39 | -4.125480757 | 3.70E-05 | 0.000627325 |
| M07 - M30 | -4.108374848 | 3.98E-05 | 0.000647486 |
| M11 - M18 | -4.111064996 | 3.94E-05 | 0.000653604 |
| M33 - M39 | -4.094496051 | 4.23E-05 | 0.000673486 |
| M16 - M19 | -4.067948886 | 4.74E-05 | 0.000739889 |
| M19 - M29 | 4.027457278 | 5.64E-05 | 0.000814426 |
| M10 - M39 | -4.028745925 | 5.61E-05 | 0.000825257 |
| M05 - M31 | -4.019132226 | 5.84E-05 | 0.000828401 |
| M17 - M24 | -4.036671873 | 5.42E-05 | 0.000829168 |
| M32 - M35 | -4.030788388 | 5.56E-05 | 0.000833851 |
| M16 - M38 | -4.012372533 | 6.01E-05 | 0.000837268 |
| M18 - M32 | 3.990229873 | 6.60E-05 | 0.000903285 |
| M07 - M20 | -3.984050526 | 6.78E-05 | 0.000911127 |
| M08 - M39 | -3.974220136 | 7.06E-05 | 0.000917933 |
| M29 - M38 | -3.969904577 | 7.19E-05 | 0.000919395 |
| M16 - M25 | -3.975463365 | 7.02E-05 | 0.000928628 |
| M19 - M33 | 3.95087377 | 7.79E-05 | 0.00096406 |
| M07 - M28 | -3.951607913 | 7.76E-05 | 0.000976608 |
| M11 - M35 | -3.926222993 | 8.63E-05 | 0.001004572 |
| M11 - M20 | -3.928259883 | 8.56E-05 | 0.001011196 |
| M25 - M29 | 3.931392284 | 8.45E-05 | 0.001013464 |
| M04 - M19 | -3.934618841 | 8.33E-05 | 0.00101557 |
| M30 - M32 | 3.901927618 | 9.54E-05 | 0.001033822 |
| M04 - M38 | -3.909765743 | 9.24E-05 | 0.00104436 |
| M33 - M38 | -3.902828231 | 9.51E-05 | 0.001044488 |
| M11 - M30 | -3.911063825 | 9.19E-05 | 0.001054038 |
| M04 - M25 | -3.903991216 | 9.46E-05 | 0.001054328 |
| M10 - M19 | -3.888745741 | 0.000100764 | 0.001076652 |
| M25 - M33 | 3.873717973 | 0.000107187 | 0.001129814 |
| M05 - M39 | -3.868340978 | 0.000109578 | 0.001139615 |
| M16 - M18 | -3.84934873 | 0.000118432 | 0.001215489 |
| M10 - M38 | -3.837078105 | 0.000124507 | 0.001261239 |
| M10 - M25 | -3.804954845 | 0.00014183 | 0.001349115 |
| M07 - M37 | -3.810889029 | 0.000138468 | 0.001350062 |
| M20 - M32 | 3.816100016 | 0.000135578 | 0.001355776 |
| M08 - M19 | -3.806046406 | 0.000141206 | 0.001359761 |
| M18 - M29 | 3.811364235 | 0.000138202 | 0.001364526 |
| M01 - M07 | 3.795935186 | 0.000147088 | 0.001365816 |
| M07 - M27 | -3.797945137 | 0.000145901 | 0.001371114 |
| M05 - M19 | -3.790443155 | 0.000150379 | 0.001379946 |
| M11 - M23 | -3.786761995 | 0.000152623 | 0.001384257 |
| M08 - M38 | -3.7650934 | 0.000166487 | 0.001492641 |
| M17 - M40 | -3.758054697 | 0.000171239 | 0.001517804 |
| M08 - M25 | -3.744310645 | 0.00018089 | 0.001585326 |
| M01 - M11 | 3.733974105 | 0.000188482 | 0.001615559 |
| M11 - M27 | -3.735593769 | 0.000187273 | 0.001623031 |
| M28 - M32 | 3.721673524 | 0.000197907 | 0.001677905 |
| M11 - M28 | -3.709870031 | 0.000207366 | 0.001702581 |
| M11 - M37 | -3.710942992 | 0.000206489 | 0.001713416 |
| M05 - M38 | -3.711844858 | 0.000205754 | 0.001725679 |
| M18 - M33 | 3.702907783 | 0.000213142 | 0.001731782 |
| M07 - M23 | -3.650376817 | 0.000261856 | 0.002063107 |
| M05 - M25 | -3.655466414 | 0.000256715 | 0.002064305 |
| M16 - M20 | -3.65201909 | 0.000260187 | 0.002070872 |
| M10 - M18 | -3.645241108 | 0.000267141 | 0.002083702 |
| M16 - M35 | -3.633698925 | 0.000279387 | 0.002136488 |
| M04 - M18 | -3.633964513 | 0.000279099 | 0.00215542 |
| M05 - M18 | -3.626305362 | 0.000287505 | 0.002177225 |
| M01 - M32 | 3.616159239 | 0.000299006 | 0.002179673 |
| M27 - M32 | 3.618146392 | 0.000296721 | 0.002183415 |
| M16 - M30 | -3.622932386 | 0.000291282 | 0.002184615 |
| M04 - M35 | -3.610244591 | 0.000305908 | 0.002189069 |
| M32 - M37 | -3.619015639 | 0.000295726 | 0.00219682 |
| M20 - M29 | 3.611527483 | 0.000304399 | 0.002198436 |
| M29 - M35 | -3.584661153 | 0.000337516 | 0.002393296 |
| M29 - M30 | -3.577532231 | 0.000346853 | 0.002437349 |
| M23 - M32 | 3.544113398 | 0.000393936 | 0.002719203 |
| M16 - M23 | -3.545869196 | 0.00039132 | 0.002725267 |
| M08 - M18 | -3.530836092 | 0.000414248 | 0.002809685 |
| M33 - M35 | -3.531570443 | 0.0004131 | 0.002826472 |
| M23 - M29 | 3.515839816 | 0.000438365 | 0.002922436 |
| M17 - M31 | -3.513346598 | 0.0004425 | 0.002924999 |
| M14 - M24 | -3.517141423 | 0.000436221 | 0.002933212 |
| M20 - M33 | 3.499331942 | 0.000466426 | 0.003057243 |
| M30 - M33 | 3.492466828 | 0.000478581 | 0.003110777 |
| M04 - M30 | -3.490166788 | 0.000482719 | 0.003111743 |
| M10 - M35 | -3.452984011 | 0.000554422 | 0.003544664 |
| M01 - M16 | 3.443301916 | 0.000574657 | 0.00361478 |
| M16 - M27 | -3.445000634 | 0.000571058 | 0.003621344 |
| M11 - M36 | -3.432716543 | 0.000597566 | 0.003641421 |
| M04 - M20 | -3.433533233 | 0.000595769 | 0.003659055 |
| M10 - M20 | -3.437203913 | 0.000587753 | 0.003667578 |
| M05 - M23 | -3.434808324 | 0.000592973 | 0.003670786 |
| M10 - M30 | -3.421150804 | 0.000623567 | 0.003741405 |
| M07 - M36 | -3.422084384 | 0.00062143 | 0.003757485 |
| M16 - M37 | -3.414879211 | 0.000638103 | 0.003770609 |
| M05 - M20 | -3.414995418 | 0.000637831 | 0.003797771 |
| M01 - M29 | 3.400833959 | 0.000671806 | 0.003853006 |
| M16 - M28 | -3.404625305 | 0.000662549 | 0.003856628 |
| M27 - M29 | 3.402532677 | 0.000667644 | 0.003857497 |
| M08 - M35 | -3.405689828 | 0.000659971 | 0.003870507 |
| M29 - M37 | -3.37080813 | 0.00074948 | 0.004236193 |
| M23 - M33 | 3.371492339 | 0.000747621 | 0.004256529 |
| M28 - M29 | 3.356314059 | 0.000789888 | 0.004432466 |
| M08 - M30 | -3.332078392 | 0.000862 | 0.00480257 |
| M10 - M23 | -3.327163613 | 0.000877348 | 0.004853417 |
| M05 - M30 | -3.324852959 | 0.000884652 | 0.004859355 |
| M08 - M20 | -3.318325489 | 0.000905589 | 0.004939576 |
| M14 - M40 | -3.300790887 | 0.000964127 | 0.005222355 |
| M11 - M21 | -3.29086959 | 0.000998782 | 0.005372759 |
| M04 - M28 | -3.278939461 | 0.00104198 | 0.00545466 |
| M05 - M35 | -3.284471758 | 0.001021737 | 0.005458597 |
| M01 - M33 | 3.279442603 | 0.001040124 | 0.005481733 |
| M27 - M33 | 3.281303455 | 0.001033285 | 0.005482737 |
| M17 - M39 | -3.271311826 | 0.001070498 | 0.00556659 |
| M28 - M33 | 3.266849156 | 0.001087516 | 0.005617633 |
| M33 - M37 | -3.254849824 | 0.001134523 | 0.005821896 |
| M32 - M36 | -3.246548847 | 0.001168134 | 0.005955193 |
| M04 - M23 | -3.241306689 | 0.001189831 | 0.006026417 |
| M06 - M24 | -3.221108612 | 0.001276957 | 0.006425979 |
| M10 - M27 | -3.215553329 | 0.001301933 | 0.006509665 |
| M01 - M10 | 3.213692477 | 0.001310399 | 0.006510264 |
| M04 - M27 | -3.210262686 | 0.001326137 | 0.006546753 |
| M01 - M04 | 3.208168366 | 0.001335833 | 0.006553142 |
| M01 - M05 | 3.202852624 | 0.001360736 | 0.006592385 |
| M05 - M27 | -3.204372004 | 0.001353575 | 0.006598676 |
| M04 - M37 | -3.198604958 | 0.001380943 | 0.006648983 |
| M10 - M28 | -3.189750299 | 0.001423958 | 0.006814031 |
| M08 - M23 | -3.184704708 | 0.001449018 | 0.006849903 |
| M10 - M37 | -3.186086696 | 0.001442114 | 0.006858834 |
| M07 - M21 | -3.17274183 | 0.001510067 | 0.007095497 |
| M06 - M40 | -3.165326041 | 0.001549093 | 0.007235283 |
| M05 - M37 | -3.1578777 | 0.001589222 | 0.007378531 |
| M17 - M19 | -3.154966108 | 0.001605168 | 0.007408465 |
| M11 - M22 | -3.139550116 | 0.001692075 | 0.007763637 |
| M11 - M26 | -3.136176028 | 0.001711664 | 0.007807592 |
| M16 - M36 | -3.127340319 | 0.001763956 | 0.007999336 |
| M08 - M28 | -3.0987684 | 0.001943269 | 0.008761558 |
| M05 - M28 | -3.095450941 | 0.001965139 | 0.008809243 |
| M29 - M36 | -3.084872363 | 0.002036395 | 0.008973945 |
| M08 - M27 | -3.086954274 | 0.002022187 | 0.009013176 |
| M01 - M08 | 3.084923917 | 0.002036042 | 0.009023369 |
| M17 - M38 | -3.066635104 | 0.00216483 | 0.009486332 |
| M08 - M37 | -3.062959237 | 0.002191599 | 0.009549985 |
| M13 - M24 | -3.050247588 | 0.002286528 | 0.009908287 |
| M03 - M24 | -3.042437211 | 0.002346708 | 0.010112884 |
| M14 - M31 | -3.038300718 | 0.002379164 | 0.010196417 |
| M21 - M32 | 3.027951333 | 0.002462177 | 0.010494526 |
| M17 - M25 | -3.004926466 | 0.002656451 | 0.011261041 |
| M07 - M22 | -2.993698215 | 0.002756184 | 0.011496383 |
| M16 - M21 | -2.996976661 | 0.002726716 | 0.011496424 |
| M24 - M34 | 2.99387394 | 0.002754597 | 0.011551537 |
| M21 - M29 | 2.958992166 | 0.003086469 | 0.012805565 |
| M06 - M31 | -2.95601481 | 0.003116421 | 0.012861421 |
| M17 - M18 | -2.941263139 | 0.003268767 | 0.01341915 |
| M33 - M36 | -2.933324015 | 0.003353537 | 0.013695073 |
| M07 - M26 | -2.930310103 | 0.003386239 | 0.013756596 |
| M13 - M40 | -2.919043777 | 0.003511069 | 0.01411667 |
| M05 - M36 | -2.920247981 | 0.00349753 | 0.014135094 |
| M03 - M40 | -2.900654846 | 0.003723838 | 0.014895352 |
| M11 - M34 | -2.878332403 | 0.003997836 | 0.015749053 |
| M16 - M26 | -2.879216959 | 0.00398664 | 0.015784665 |
| M06 - M39 | -2.880794124 | 0.003966747 | 0.015786032 |
| M07 - M34 | -2.871167986 | 0.004089581 | 0.016029515 |
| M10 - M36 | -2.867573889 | 0.004136322 | 0.016131656 |
| M05 - M21 | -2.848199679 | 0.004396733 | 0.016811037 |
| M26 - M29 | 2.849187579 | 0.004383103 | 0.01684148 |
| M22 - M32 | 2.850418661 | 0.004366172 | 0.016859475 |
| M06 - M19 | -2.851898606 | 0.004345896 | 0.016864671 |
| M14 - M39 | -2.845171547 | 0.004438751 | 0.016888907 |
| M16 - M22 | -2.839720851 | 0.004515303 | 0.017096777 |
| M26 - M32 | 2.827483776 | 0.004691539 | 0.017678264 |
| M34 - M40 | -2.822437703 | 0.004766008 | 0.01787253 |
| M04 - M36 | -2.818624748 | 0.004822987 | 0.017999663 |
| M05 - M26 | -2.806282568 | 0.005011672 | 0.018614783 |
| M22 - M29 | 2.801736356 | 0.005082839 | 0.018789643 |
| M21 - M33 | 2.787313307 | 0.005314707 | 0.01955411 |
| M09 - M24 | -2.78475087 | 0.005356886 | 0.019616767 |
| M17 - M23 | -2.754479927 | 0.00587855 | 0.02113027 |
| M14 - M19 | -2.758434672 | 0.005807891 | 0.021168949 |
| M06 - M23 | -2.754668685 | 0.00587516 | 0.021215854 |
| M06 - M18 | -2.755058659 | 0.005868161 | 0.021289144 |
| M06 - M38 | -2.737933366 | 0.00618266 | 0.022121445 |
| M10 - M21 | -2.729646632 | 0.006340225 | 0.022581622 |
| M09 - M40 | -2.714007518 | 0.006647466 | 0.023568288 |
| M08 - M36 | -2.707277577 | 0.006783751 | 0.023942651 |
| M05 - M22 | -2.704645422 | 0.006837733 | 0.024024468 |
| M02 - M11 | 2.688058132 | 0.007186888 | 0.025137995 |
| M26 - M33 | 2.675196787 | 0.00746854 | 0.025890938 |
| M17 - M20 | -2.676362274 | 0.007442615 | 0.025916248 |
| M13 - M31 | -2.655395369 | 0.00792155 | 0.027339864 |
| M06 - M25 | -2.653676161 | 0.00796202 | 0.027358482 |
| M32 - M34 | -2.64739484 | 0.008111458 | 0.027749726 |
| M14 - M38 | -2.643135926 | 0.008214205 | 0.027978516 |
| M04 - M21 | -2.633811454 | 0.008443236 | 0.028633583 |
| M10 - M26 | -2.630868061 | 0.008516709 | 0.028633764 |
| M03 - M31 | -2.631628588 | 0.00849767 | 0.028693432 |
| M15 - M24 | -2.62093016 | 0.008769022 | 0.029355526 |
| M22 - M33 | 2.618393471 | 0.008834487 | 0.02944829 |
| M15 - M40 | -2.611742939 | 0.009008196 | 0.029899544 |
| M14 - M18 | -2.580353039 | 0.009869936 | 0.032620974 |
| M17 - M30 | -2.567203442 | 0.010252244 | 0.033741563 |
| M10 - M22 | -2.560726795 | 0.010445346 | 0.034232647 |
| M14 - M25 | -2.558411215 | 0.010515167 | 0.034317281 |
| M08 - M21 | -2.553098273 | 0.010676937 | 0.034700044 |
| M31 - M34 | 2.537235107 | 0.011173189 | 0.036162188 |
| M17 - M35 | -2.532564217 | 0.011323163 | 0.036496144 |
| M13 - M39 | -2.524702632 | 0.011579623 | 0.037169159 |
| M16 - M34 | -2.515735248 | 0.011878437 | 0.037972054 |
| M04 - M26 | -2.509096341 | 0.012104047 | 0.038535332 |
| M06 - M20 | -2.507027838 | 0.012175112 | 0.038604014 |
| M03 - M39 | -2.494162797 | 0.012625462 | 0.039869881 |
| M14 - M23 | -2.487963716 | 0.012847684 | 0.040245757 |
| M12 - M24 | -2.487984427 | 0.012846936 | 0.040405685 |
| M12 - M40 | -2.483305159 | 0.013016952 | 0.040612891 |
| M02 - M16 | 2.473400456 | 0.013383408 | 0.041589874 |
| M13 - M19 | -2.471897623 | 0.0134398 | 0.04159938 |
| M29 - M34 | -2.466056432 | 0.013660981 | 0.04178653 |
| M09 - M31 | -2.466478493 | 0.013644892 | 0.041901638 |
| M03 - M11 | 2.467068875 | 0.013622415 | 0.04199796 |
| M02 - M05 | 2.460586218 | 0.013871024 | 0.042098827 |
| M08 - M26 | -2.461618833 | 0.013831157 | 0.042141807 |
| M04 - M22 | -2.449291237 | 0.014313766 | 0.042941298 |
| M02 - M07 | 2.449828352 | 0.014292433 | 0.043042848 |
| M02 - M29 | 2.450700379 | 0.014257858 | 0.043105152 |
| M03 - M19 | -2.438700982 | 0.014740159 | 0.044051051 |
| M15 - M23 | -2.424427461 | 0.015332546 | 0.045646511 |
| M15 - M31 | -2.417247555 | 0.015638377 | 0.046379979 |
| M17 - M27 | -2.402926121 | 0.016264473 | 0.048054125 |
| M01 - M17 | 2.400938968 | 0.016353064 | 0.048133546 |
| M09 - M39 | -2.381230471 | 0.017254913 | 0.049847525 |
| M15 - M19 | -2.381496307 | 0.017242464 | 0.049996735 |
| M02 - M32 | 2.378547245 | 0.017381009 | 0.050026522 |
| M11 - M14 | -2.382192363 | 0.017209905 | 0.050088529 |
| M15 - M39 | -2.384332776 | 0.017110122 | 0.050172539 |
| M34 - M39 | -2.382596021 | 0.017191048 | 0.050221039 |
| M08 - M22 | -2.372713488 | 0.017657955 | 0.050636783 |
| M09 - M19 | -2.352117713 | 0.018666867 | 0.053333904 |
| M17 - M37 | -2.339663196 | 0.019301137 | 0.054944843 |
| M13 - M38 | -2.337653992 | 0.019405205 | 0.055040217 |
| M13 - M18 | -2.336285768 | 0.019476353 | 0.055041867 |
| M11 - M13 | -2.327903587 | 0.019917224 | 0.055682561 |
| M15 - M18 | -2.3255453 | 0.020042821 | 0.055833572 |
| M06 - M30 | -2.328015584 | 0.019911276 | 0.055866171 |
| M13 - M23 | -2.328857055 | 0.01986664 | 0.055942162 |
| M12 - M23 | -2.321448139 | 0.02026267 | 0.056045683 |
| M19 - M34 | 2.321452241 | 0.020262449 | 0.05624452 |
| M05 - M34 | -2.310497671 | 0.020860617 | 0.057495694 |
| M03 - M38 | -2.302494977 | 0.021307274 | 0.058519979 |
| M03 - M18 | -2.299322669 | 0.021486624 | 0.058805496 |
| M09 - M23 | -2.29428262 | 0.02177427 | 0.059384373 |
| M03 - M23 | -2.292547879 | 0.021874048 | 0.059448632 |
| M12 - M31 | -2.286987871 | 0.022196531 | 0.059907592 |
| M02 - M33 | 2.28822844 | 0.022124221 | 0.059919766 |
| M14 - M20 | -2.285275882 | 0.022296656 | 0.059970317 |
| M06 - M27 | -2.27467615 | 0.022925364 | 0.06144943 |
| M01 - M06 | 2.273289152 | 0.023008761 | 0.061461758 |
| M03 - M07 | 2.263934494 | 0.023578144 | 0.06255426 |
| M33 - M34 | -2.264753775 | 0.023527794 | 0.062633717 |
| M17 - M28 | -2.257653886 | 0.023967246 | 0.062733061 |
| M12 - M39 | -2.25820938 | 0.023932609 | 0.062853316 |
| M15 - M38 | -2.26061173 | 0.023783311 | 0.062884686 |
| M02 - M10 | 2.255353377 | 0.024111157 | 0.06289867 |
| M12 - M19 | -2.258412475 | 0.023919956 | 0.063032316 |
| M09 - M18 | -2.24961617 | 0.024473321 | 0.063630635 |
| M13 - M25 | -2.236843938 | 0.025296542 | 0.0655525 |
| M06 - M35 | -2.225131721 | 0.026072397 | 0.067339303 |
| M09 - M38 | -2.214405047 | 0.026800928 | 0.068992488 |
| M12 - M18 | -2.206635021 | 0.027339568 | 0.070147576 |
| M06 - M37 | -2.201718768 | 0.02768518 | 0.070801444 |
| M03 - M25 | -2.200049394 | 0.027803391 | 0.070871388 |
| M07 - M14 | -2.18407834 | 0.028956492 | 0.073331376 |
| M10 - M34 | -2.184839028 | 0.028900651 | 0.073428365 |
| M23 - M34 | 2.177004581 | 0.029480226 | 0.074176052 |
| M34 - M38 | -2.177919298 | 0.029412045 | 0.074243998 |
| M18 - M34 | 2.174131995 | 0.029695224 | 0.074476767 |
| M15 - M25 | -2.171384836 | 0.029902094 | 0.074755236 |
| M06 - M26 | -2.158396797 | 0.030896995 | 0.076995705 |
| M12 - M38 | -2.134488334 | 0.032802828 | 0.081484731 |
| M02 - M04 | 2.130599566 | 0.033122145 | 0.082016741 |
| M09 - M25 | -2.111022352 | 0.034770391 | 0.085825648 |
| M14 - M30 | -2.106827337 | 0.035132549 | 0.08644602 |
| M02 - M08 | 2.104720872 | 0.035315613 | 0.086623201 |
| M04 - M34 | -2.100388561 | 0.035694674 | 0.087278514 |
| M03 - M16 | 2.092838915 | 0.036363537 | 0.088636121 |
| M15 - M20 | -2.080468421 | 0.037482589 | 0.091079189 |
| M03 - M32 | 2.074999746 | 0.037986558 | 0.092017129 |
| M07 - M13 | -2.071969517 | 0.038268284 | 0.092412574 |
| M06 - M28 | -2.068630578 | 0.038580766 | 0.092593839 |
| M25 - M34 | 2.069309472 | 0.038517055 | 0.092726244 |
| M03 - M29 | 2.046317599 | 0.040725133 | 0.097440503 |
| M11 - M17 | -2.044818537 | 0.040872752 | 0.097494637 |
| M12 - M25 | -2.042947056 | 0.041057679 | 0.097637165 |
| M17 - M26 | -2.037850305 | 0.041564906 | 0.098542938 |
| M06 - M21 | -2.034672778 | 0.041883809 | 0.098998094 |
| M13 - M20 | -2.030109469 | 0.042345413 | 0.099486212 |
| M17 - M36 | -2.031328576 | 0.042221674 | 0.099495184 |
| M06 - M36 | -2.015307589 | 0.04387243 | 0.102764251 |
| M14 - M35 | -2.013169638 | 0.044096786 | 0.102980519 |
| M14 - M16 | 1.989952799 | 0.046596135 | 0.108492494 |
| M01 - M14 | 1.986029777 | 0.047030018 | 0.10853081 |
| M03 - M20 | -1.987159155 | 0.046904763 | 0.108562952 |
| M14 - M27 | -1.987991288 | 0.046812654 | 0.108672231 |
| M02 - M06 | 1.984102929 | 0.047244367 | 0.108703852 |
| M14 - M32 | 1.977750134 | 0.047956902 | 0.109696138 |
| M17 - M21 | -1.978984599 | 0.04781774 | 0.109699522 |
| M09 - M11 | 1.974364631 | 0.048340297 | 0.1102498 |
| M03 - M05 | 1.967739286 | 0.049098043 | 0.111651527 |
| M08 - M34 | -1.962896237 | 0.049658232 | 0.112597154 |
| M12 - M20 | -1.95738459 | 0.050302269 | 0.113726869 |
| M09 - M20 | -1.953895252 | 0.050713606 | 0.114325471 |
| M13 - M16 | 1.951752673 | 0.050967576 | 0.114566885 |
| M14 - M29 | 1.940915027 | 0.052268585 | 0.117153725 |
| M13 - M29 | 1.906352518 | 0.056604485 | 0.126508591 |
| M06 - M22 | -1.901767362 | 0.057201575 | 0.127114611 |
| M14 - M37 | -1.902642505 | 0.057087209 | 0.127222923 |
| M13 - M32 | 1.893419568 | 0.058302095 | 0.129192143 |
| M15 - M30 | -1.879461147 | 0.060181554 | 0.132603424 |
| M15 - M26 | -1.880108189 | 0.060093335 | 0.132784139 |
| M05 - M14 | -1.860945855 | 0.062751823 | 0.137877246 |
| M01 - M15 | 1.858218037 | 0.063138058 | 0.137948697 |
| M15 - M27 | -1.859419213 | 0.062967741 | 0.137963027 |
| M05 - M13 | -1.851010986 | 0.064167973 | 0.139807315 |
| M20 - M34 | 1.842848407 | 0.065351147 | 0.141988564 |
| M13 - M30 | -1.805080397 | 0.071062102 | 0.153967888 |
| M02 - M17 | 1.800594724 | 0.071766781 | 0.154635606 |
| M17 - M22 | -1.801451927 | 0.071631678 | 0.154772046 |
| M02 - M15 | 1.796958102 | 0.072342272 | 0.155446203 |
| M03 - M33 | 1.789226972 | 0.07357827 | 0.157667721 |
| M15 - M37 | -1.782401845 | 0.074683725 | 0.159598097 |
| M12 - M26 | -1.777128867 | 0.07554704 | 0.161001888 |
| M14 - M26 | -1.775282496 | 0.075851254 | 0.16120975 |
| M24 - M28 | 1.769187392 | 0.076862608 | 0.16291531 |
| M15 - M35 | -1.762441393 | 0.07799475 | 0.164866952 |
| M14 - M28 | -1.755857732 | 0.079112704 | 0.166328596 |
| M03 - M30 | -1.756661701 | 0.078975489 | 0.166488869 |
| M12 - M30 | -1.749201464 | 0.080256201 | 0.168279131 |
| M28 - M40 | -1.730948483 | 0.083460953 | 0.172677834 |
| M01 - M13 | 1.729291912 | 0.083756863 | 0.172831623 |
| M13 - M27 | -1.731107918 | 0.083432518 | 0.173078097 |
| M01 - M12 | 1.732094641 | 0.083256713 | 0.173173963 |
| M07 - M17 | -1.732371246 | 0.083207484 | 0.173534325 |
| M12 - M27 | -1.733295816 | 0.083043104 | 0.173655821 |
| M09 - M30 | -1.720702929 | 0.085304745 | 0.175561216 |
| M03 - M10 | 1.715716096 | 0.086214017 | 0.176965614 |
| M02 - M12 | 1.712875838 | 0.086735384 | 0.177568503 |
| M15 - M21 | -1.69022467 | 0.09098498 | 0.18578085 |
| M03 - M27 | -1.680970201 | 0.092768703 | 0.188928429 |
| M01 - M03 | 1.679109348 | 0.093130735 | 0.189171805 |
| M13 - M35 | -1.671172831 | 0.094687545 | 0.190843114 |
| M09 - M27 | -1.673437167 | 0.094241266 | 0.190930358 |
| M01 - M09 | 1.671817503 | 0.094560314 | 0.191080428 |
| M14 - M33 | 1.667737527 | 0.095367842 | 0.191718858 |
| M12 - M37 | -1.653964065 | 0.098134814 | 0.196774178 |
| M09 - M26 | -1.643696653 | 0.100238878 | 0.200477755 |
| M13 - M26 | -1.640341586 | 0.100934164 | 0.20135204 |
| M13 - M37 | -1.63423408 | 0.102209709 | 0.201831831 |
| M15 - M36 | -1.63479945 | 0.102091096 | 0.202109277 |
| M13 - M33 | 1.635054423 | 0.102037639 | 0.202517452 |
| M15 - M28 | -1.635410644 | 0.101962993 | 0.202885547 |
| M16 - M17 | -1.627019442 | 0.103732969 | 0.203295768 |
| M14 - M21 | -1.627372094 | 0.103658095 | 0.20366074 |
| M12 - M35 | -1.627609811 | 0.103607647 | 0.204075669 |
| M14 - M36 | -1.621188751 | 0.104977164 | 0.205218517 |
| M03 - M35 | -1.618806353 | 0.105488935 | 0.205703423 |
| M02 - M14 | 1.610826554 | 0.107217532 | 0.207517803 |
| M09 - M16 | 1.611242057 | 0.107126974 | 0.207858308 |
| M07 - M09 | -1.612111652 | 0.106937646 | 0.208008388 |
| M24 - M35 | 1.597659487 | 0.11011878 | 0.212605565 |
| M03 - M26 | -1.596252327 | 0.110432469 | 0.212684755 |
| M30 - M34 | 1.591091951 | 0.111588884 | 0.214382584 |
| M10 - M14 | -1.589151096 | 0.112026284 | 0.214694108 |
| M09 - M35 | -1.580328247 | 0.114031714 | 0.217468795 |
| M03 - M37 | -1.581181245 | 0.113836602 | 0.217628799 |
| M17 - M29 | 1.577340626 | 0.114717169 | 0.217711416 |
| M09 - M37 | -1.578385455 | 0.114477085 | 0.217785673 |
| M09 - M29 | 1.57075045 | 0.116240626 | 0.217951173 |
| M35 - M40 | -1.572695984 | 0.11578923 | 0.218153622 |
| M12 - M21 | -1.571314392 | 0.11610964 | 0.218230167 |
| M05 - M09 | -1.572845308 | 0.115754642 | 0.218616514 |
| M15 - M22 | -1.57301311 | 0.115715783 | 0.219073569 |
| M10 - M13 | -1.563738398 | 0.117879016 | 0.220493124 |
| M02 - M09 | 1.548158181 | 0.121584217 | 0.22687964 |
| M05 - M17 | -1.543366527 | 0.12274187 | 0.22849322 |
| M17 - M32 | 1.529118641 | 0.126235035 | 0.234436493 |
| M02 - M13 | 1.525003919 | 0.12725812 | 0.235216431 |
| M03 - M04 | 1.525743589 | 0.127073734 | 0.235433522 |
| M23 - M28 | 1.518496181 | 0.128889362 | 0.237668326 |
| M27 - M34 | 1.514210315 | 0.129972506 | 0.239100365 |
| M01 - M34 | 1.512223162 | 0.1304771 | 0.239463855 |
| M12 - M36 | -1.508676054 | 0.131381588 | 0.23999447 |
| M04 - M11 | 1.509418723 | 0.131191811 | 0.240210359 |
| M12 - M28 | -1.501446155 | 0.133240204 | 0.242820932 |
| M08 - M11 | 1.492790656 | 0.135491994 | 0.24634908 |
| M02 - M03 | 1.488061813 | 0.136734593 | 0.248030192 |
| M09 - M32 | 1.467810177 | 0.142155792 | 0.257265703 |
| M26 - M34 | 1.460374959 | 0.144187051 | 0.260337731 |
| M12 - M22 | -1.454102832 | 0.145917799 | 0.262854233 |
| M14 - M22 | -1.451554754 | 0.146625447 | 0.263520389 |
| M13 - M28 | -1.450190649 | 0.147005362 | 0.263595821 |
| M34 - M35 | -1.436881785 | 0.150751586 | 0.269076058 |
| M13 - M21 | -1.437806717 | 0.150488903 | 0.269223267 |
| M03 - M08 | 1.435337655 | 0.151190902 | 0.269244073 |
| M09 - M21 | -1.429420764 | 0.152883334 | 0.271637814 |
| M23 - M35 | 1.41187928 | 0.15798551 | 0.280065221 |
| M28 - M31 | -1.407161275 | 0.159379572 | 0.281895841 |
| M34 - M37 | -1.404046201 | 0.160305092 | 0.282891339 |
| M09 - M28 | -1.394722286 | 0.163099602 | 0.287173114 |
| M13 - M36 | -1.391514757 | 0.164069388 | 0.288230006 |
| M03 - M28 | -1.390292077 | 0.164440203 | 0.288232266 |
| M03 - M21 | -1.383728193 | 0.166441697 | 0.291086377 |
| M04 - M14 | -1.381340798 | 0.1671742 | 0.291713368 |
| M02 - M34 | 1.377926049 | 0.168226121 | 0.292893693 |
| M09 - M36 | -1.370559941 | 0.170512178 | 0.296212692 |
| M36 - M40 | -1.364692511 | 0.172349715 | 0.298739505 |
| M19 - M28 | 1.363380212 | 0.172762714 | 0.29879139 |
| M28 - M39 | -1.358863609 | 0.174189812 | 0.300593039 |
| M04 - M13 | -1.348724048 | 0.177425618 | 0.305501064 |
| M24 - M36 | 1.34655035 | 0.178125094 | 0.306029897 |
| M06 - M34 | -1.344397067 | 0.178820022 | 0.306548609 |
| M23 - M36 | 1.334505649 | 0.182038197 | 0.311381126 |
| M03 - M36 | -1.332990761 | 0.182534836 | 0.311547422 |
| M18 - M28 | 1.296222945 | 0.194898687 | 0.331923528 |
| M08 - M14 | -1.283622703 | 0.199274002 | 0.337899395 |
| M09 - M33 | 1.283822974 | 0.199203903 | 0.338516436 |
| M09 - M22 | -1.278101291 | 0.201213714 | 0.340448366 |
| M13 - M22 | -1.272044539 | 0.203357295 | 0.343330498 |
| M08 - M13 | -1.270531303 | 0.20389544 | 0.343495557 |
| M17 - M33 | 1.253234894 | 0.210120235 | 0.35321936 |
| M37 - M40 | -1.244011641 | 0.213495279 | 0.358121113 |
| M23 - M37 | 1.241390461 | 0.214461541 | 0.358969962 |
| M31 - M35 | 1.237667054 | 0.21583954 | 0.360502872 |
| M22 - M23 | -1.226474585 | 0.220020134 | 0.366700223 |
| M09 - M10 | 1.221694945 | 0.221823009 | 0.368916731 |
| M24 - M37 | 1.219676173 | 0.222587658 | 0.369400794 |
| M19 - M35 | 1.214404043 | 0.224593472 | 0.37115023 |
| M03 - M22 | -1.214808357 | 0.224439193 | 0.37168274 |
| M21 - M34 | 1.211853455 | 0.225568482 | 0.371973395 |
| M35 - M39 | -1.201425421 | 0.22958622 | 0.377800109 |
| M28 - M34 | 1.186985939 | 0.235233167 | 0.385466114 |
| M23 - M30 | 1.187223972 | 0.235139288 | 0.386123462 |
| M30 - M40 | -1.175170438 | 0.239926535 | 0.392332698 |
| M10 - M17 | -1.173320147 | 0.240667446 | 0.392720937 |
| M22 - M40 | -1.167522845 | 0.242999288 | 0.395698215 |
| M28 - M38 | -1.159821273 | 0.246121581 | 0.399947569 |
| M18 - M35 | 1.156827136 | 0.247342993 | 0.401096745 |
| M24 - M30 | 1.147525783 | 0.251164383 | 0.406448587 |
| M34 - M36 | -1.142612771 | 0.253199401 | 0.408893443 |
| M22 - M24 | -1.128947686 | 0.258919903 | 0.417267612 |
| M17 - M34 | -1.118276199 | 0.263449049 | 0.423691254 |
| M21 - M23 | -1.109263025 | 0.267316731 | 0.427268546 |
| M23 - M27 | 1.109885887 | 0.267048203 | 0.427715808 |
| M01 - M23 | -1.111087062 | 0.266530876 | 0.427765604 |
| M19 - M36 | 1.086146888 | 0.277414011 | 0.442500877 |
| M03 - M06 | 1.083734767 | 0.278482416 | 0.443298539 |
| M31 - M36 | 1.066903648 | 0.286015348 | 0.454362467 |
| M36 - M39 | -1.060000195 | 0.289144511 | 0.458399834 |
| M18 - M36 | 1.052170513 | 0.292721325 | 0.463129074 |
| M22 - M34 | 1.034320783 | 0.300986224 | 0.472372746 |
| M01 - M40 | -1.036803666 | 0.299827367 | 0.472455244 |
| M27 - M40 | -1.035040823 | 0.300649847 | 0.47279613 |
| M04 - M16 | 1.037019343 | 0.299726842 | 0.473252909 |
| M08 - M16 | 1.030609043 | 0.302724194 | 0.474146327 |
| M04 - M05 | 1.027176615 | 0.304337308 | 0.475717635 |
| M05 - M08 | -1.025840428 | 0.304966806 | 0.475748218 |
| M10 - M11 | 1.017024117 | 0.309141957 | 0.481298855 |
| M15 - M34 | -1.014902491 | 0.31015231 | 0.481909964 |
| M11 - M12 | -1.012100763 | 0.311489877 | 0.483026052 |
| M21 - M40 | -1.005571801 | 0.314621594 | 0.486914372 |
| M25 - M28 | 1.000697182 | 0.31697323 | 0.489582415 |
| M35 - M38 | -0.999389799 | 0.3176059 | 0.489590122 |
| M01 - M24 | -0.993294546 | 0.320566435 | 0.491241295 |
| M24 - M27 | 0.991395321 | 0.321492584 | 0.49169454 |
| M06 - M13 | -0.994875359 | 0.319796889 | 0.491995213 |
| M04 - M07 | 0.99330675 | 0.320560489 | 0.492199176 |
| M04 - M29 | 0.98466133 | 0.324790453 | 0.49576625 |
| M08 - M29 | 0.979850127 | 0.327160104 | 0.498407972 |
| M07 - M08 | -0.974315157 | 0.329900081 | 0.500626582 |
| M04 - M09 | -0.974935624 | 0.329592194 | 0.50113433 |
| M19 - M37 | 0.962192043 | 0.335953145 | 0.508822239 |
| M21 - M24 | -0.957367531 | 0.338381781 | 0.511507343 |
| M11 - M33 | -0.954896087 | 0.339630249 | 0.512401536 |
| M06 - M14 | -0.951891446 | 0.341152038 | 0.513703841 |
| M19 - M22 | 0.939496556 | 0.34747586 | 0.522218055 |
| M18 - M37 | 0.93314649 | 0.350744339 | 0.526116508 |
| M37 - M39 | -0.930529402 | 0.352097048 | 0.526122026 |
| M31 - M37 | 0.931121567 | 0.351790684 | 0.526673193 |
| M08 - M09 | -0.9253023 | 0.354808683 | 0.529160177 |
| M18 - M22 | 0.92165994 | 0.356705982 | 0.530974554 |
| M11 - M32 | -0.905080795 | 0.365422586 | 0.541881401 |
| M02 - M28 | 0.903490507 | 0.366265624 | 0.542100924 |
| M22 - M39 | -0.905550362 | 0.365173892 | 0.542544068 |
| M06 - M11 | 0.894945014 | 0.370816482 | 0.545729918 |
| M22 - M31 | -0.896076701 | 0.3702118 | 0.545869951 |
| M20 - M28 | 0.896422985 | 0.370026896 | 0.546630641 |
| M19 - M30 | 0.888737385 | 0.374144234 | 0.548557335 |
| M11 - M15 | -0.889016932 | 0.37399398 | 0.549369688 |
| M36 - M38 | -0.885032214 | 0.376139257 | 0.550447693 |
| M04 - M17 | -0.881775326 | 0.377898317 | 0.551986306 |
| M12 - M34 | -0.879323914 | 0.379225673 | 0.552889766 |
| M18 - M30 | 0.862443795 | 0.388443349 | 0.565272038 |
| M23 - M25 | 0.852407471 | 0.393987975 | 0.571209332 |
| M30 - M39 | -0.853522918 | 0.393369384 | 0.571374524 |
| M30 - M31 | -0.850314971 | 0.395149995 | 0.571831162 |
| M25 - M35 | 0.830316312 | 0.406359968 | 0.586964398 |
| M02 - M35 | 0.821196456 | 0.411534375 | 0.593339764 |
| M20 - M23 | -0.817276642 | 0.413770352 | 0.595462869 |
| M06 - M09 | -0.814904913 | 0.415126741 | 0.596314655 |
| M26 - M28 | 0.810398168 | 0.417711371 | 0.598924392 |
| M03 - M15 | 0.808790149 | 0.418635863 | 0.599148575 |
| M08 - M17 | -0.805370401 | 0.420605967 | 0.600865667 |
| M02 - M36 | 0.801766741 | 0.422687901 | 0.602735946 |
| M19 - M27 | 0.783269661 | 0.433468775 | 0.612510226 |
| M04 - M32 | 0.784543082 | 0.432721513 | 0.612564029 |
| M19 - M21 | 0.788177083 | 0.430593132 | 0.612888035 |
| M07 - M11 | 0.784676105 | 0.432643496 | 0.61356714 |
| M01 - M19 | -0.784889326 | 0.432518461 | 0.614507103 |
| M08 - M32 | 0.777417765 | 0.436912335 | 0.615147331 |
| M18 - M21 | 0.778105683 | 0.436506713 | 0.615687588 |
| M01 - M18 | -0.76956587 | 0.441557457 | 0.620567237 |
| M18 - M27 | 0.76804649 | 0.442459565 | 0.620716656 |
| M05 - M12 | -0.754230908 | 0.450710581 | 0.631156649 |
| M21 - M39 | -0.748294553 | 0.454282509 | 0.63162274 |
| M37 - M38 | -0.748956547 | 0.453883396 | 0.632194731 |
| M22 - M38 | -0.749054243 | 0.453824513 | 0.633243506 |
| M02 - M22 | 0.750018259 | 0.453243708 | 0.633566473 |
| M01 - M39 | -0.744038598 | 0.456853124 | 0.634066614 |
| M20 - M35 | 0.741245253 | 0.45854475 | 0.634157632 |
| M27 - M39 | -0.74233988 | 0.457881435 | 0.634365043 |
| M13 - M15 | 0.737828064 | 0.460618937 | 0.635898709 |
| M01 - M31 | -0.729126493 | 0.46592429 | 0.63982561 |
| M25 - M36 | 0.73006894 | 0.465348045 | 0.640161332 |
| M27 - M31 | -0.727310487 | 0.467035776 | 0.640224789 |
| M21 - M31 | -0.730314523 | 0.465197953 | 0.641085518 |
| M02 - M37 | 0.721872747 | 0.470372718 | 0.64366793 |
| M23 - M38 | 0.708693369 | 0.478514781 | 0.653662923 |
| M12 - M16 | 0.702687493 | 0.482250524 | 0.65761435 |
| M26 - M35 | 0.699198061 | 0.484428262 | 0.659431142 |
| M03 - M17 | 0.691859893 | 0.48902532 | 0.66452918 |
| M14 - M15 | 0.686356958 | 0.49248804 | 0.668070732 |
| M03 - M12 | 0.677058558 | 0.498368801 | 0.674874418 |
| M02 - M30 | 0.674080859 | 0.500259908 | 0.676261227 |
| M12 - M29 | 0.672658113 | 0.501164821 | 0.676312389 |
| M20 - M36 | 0.670217092 | 0.50271941 | 0.677238583 |
| M26 - M36 | 0.667853412 | 0.504227168 | 0.678098605 |
| M30 - M38 | -0.666474279 | 0.505107999 | 0.678114009 |
| M02 - M21 | 0.659226575 | 0.509750284 | 0.680830859 |
| M06 - M17 | -0.659595547 | 0.509513413 | 0.681681753 |
| M25 - M40 | -0.660537154 | 0.508909184 | 0.682043236 |
| M23 - M31 | 0.649437565 | 0.51605559 | 0.68807412 |
| M05 - M15 | -0.63532063 | 0.525219322 | 0.69909739 |
| M02 - M27 | 0.631970161 | 0.527406376 | 0.699620702 |
| M01 - M02 | -0.632878164 | 0.526813208 | 0.700024366 |
| M05 - M10 | -0.621820847 | 0.534059677 | 0.707243714 |
| M14 - M34 | -0.616156469 | 0.537791256 | 0.71097827 |
| M05 - M06 | -0.602247733 | 0.547009247 | 0.717087752 |
| M09 - M15 | 0.603462443 | 0.546201093 | 0.717233759 |
| M12 - M13 | -0.607568381 | 0.543473777 | 0.717275036 |
| M20 - M40 | -0.603559437 | 0.546136588 | 0.718358412 |
| M22 - M25 | -0.604214867 | 0.545700798 | 0.718997673 |
| M22 - M26 | -0.597948828 | 0.549874078 | 0.719633861 |
| M21 - M38 | -0.591798433 | 0.553985573 | 0.723800246 |
| M24 - M25 | 0.586809236 | 0.557331833 | 0.726954565 |
| M23 - M39 | 0.584972323 | 0.558566327 | 0.727348472 |
| M25 - M37 | 0.583474486 | 0.559573928 | 0.727446106 |
| M07 - M12 | -0.579389588 | 0.562326328 | 0.72980788 |
| M15 - M16 | 0.576564097 | 0.564233959 | 0.731067255 |
| M20 - M22 | 0.564048819 | 0.572720908 | 0.735951084 |
| M26 - M37 | 0.562505053 | 0.573771962 | 0.73608903 |
| M10 - M16 | 0.558255786 | 0.576669732 | 0.736174126 |
| M01 - M38 | -0.569070618 | 0.569308217 | 0.73641859 |
| M27 - M38 | -0.567371899 | 0.570461538 | 0.736688741 |
| M04 - M33 | 0.558642753 | 0.576405556 | 0.73704317 |
| M05 - M33 | -0.564154172 | 0.572649214 | 0.737073245 |
| M08 - M33 | 0.565078913 | 0.572020091 | 0.737480448 |
| M13 - M17 | 0.558695393 | 0.576369624 | 0.7382074 |
| M12 - M14 | -0.551525376 | 0.581273576 | 0.740838872 |
| M15 - M29 | 0.546534717 | 0.58469843 | 0.743988215 |
| M23 - M26 | 0.544319272 | 0.586221787 | 0.744711717 |
| M06 - M16 | 0.538155089 | 0.590469983 | 0.748888759 |
| M20 - M37 | 0.532062625 | 0.594682622 | 0.753007217 |
| M20 - M24 | -0.528586971 | 0.597092001 | 0.754832675 |
| M19 - M23 | -0.516248756 | 0.605680681 | 0.764451345 |
| M10 - M29 | 0.511734471 | 0.608836858 | 0.764722623 |
| M27 - M28 | 0.514383018 | 0.60698423 | 0.764858965 |
| M01 - M28 | 0.512450568 | 0.608335707 | 0.765325567 |
| M23 - M24 | 0.508992234 | 0.610757668 | 0.765901898 |
| M09 - M34 | -0.505441432 | 0.613248825 | 0.767791466 |
| M06 - M29 | 0.503480148 | 0.614626734 | 0.768283418 |
| M26 - M30 | 0.498708502 | 0.617984754 | 0.771244973 |
| M11 - M29 | -0.491406152 | 0.623139214 | 0.771505693 |
| M28 - M30 | -0.492175451 | 0.622595317 | 0.772057786 |
| M16 - M33 | -0.49250566 | 0.622361921 | 0.772997291 |
| M12 - M32 | 0.487784903 | 0.625702206 | 0.773451222 |
| M05 - M32 | -0.494399793 | 0.62102386 | 0.773799698 |
| M25 - M30 | 0.492970598 | 0.622033362 | 0.773821408 |
| M14 - M17 | 0.479525963 | 0.631564503 | 0.775780019 |
| M09 - M12 | 0.480378611 | 0.6309582 | 0.776257723 |
| M08 - M10 | 0.482891255 | 0.629172951 | 0.776510921 |
| M21 - M26 | -0.480737268 | 0.63070324 | 0.77716987 |
| M04 - M10 | 0.473005175 | 0.636209487 | 0.779032025 |
| M18 - M23 | -0.473942395 | 0.635540986 | 0.779437058 |
| M21 - M25 | -0.442263823 | 0.658298306 | 0.796081673 |
| M07 - M15 | -0.44316075 | 0.657649467 | 0.796531963 |
| M11 - M16 | -0.450914544 | 0.652051141 | 0.79717851 |
| M26 - M27 | 0.44323365 | 0.657596742 | 0.797706779 |
| M29 - M33 | -0.445984345 | 0.655608554 | 0.7977764 |
| M01 - M26 | -0.444434825 | 0.65672824 | 0.797894124 |
| M38 - M40 | -0.446251177 | 0.655415819 | 0.798788029 |
| M15 - M17 | -0.437427145 | 0.661801601 | 0.799079333 |
| M20 - M30 | 0.446949231 | 0.654911717 | 0.799422753 |
| M18 - M25 | 0.435557776 | 0.663157612 | 0.799479037 |
| M19 - M25 | 0.429555147 | 0.667519271 | 0.802257367 |
| M02 - M25 | 0.430135504 | 0.667097075 | 0.80298722 |
| M02 - M20 | 0.420076231 | 0.674429766 | 0.809315719 |
| M13 - M34 | -0.417416098 | 0.676374082 | 0.810402126 |
| M16 - M32 | -0.411799171 | 0.680486629 | 0.811589558 |
| M23 - M40 | 0.412049368 | 0.680303239 | 0.812613364 |
| M20 - M21 | 0.412729346 | 0.679804922 | 0.813263557 |
| M18 - M20 | 0.40746606 | 0.683665717 | 0.814136274 |
| M12 - M33 | 0.401885902 | 0.687767999 | 0.816528218 |
| M25 - M27 | 0.400417253 | 0.688849219 | 0.816568983 |
| M19 - M20 | 0.398222461 | 0.690466211 | 0.81724377 |
| M01 - M25 | -0.402180096 | 0.68755149 | 0.817515491 |
| M06 - M07 | 0.384729756 | 0.700437647 | 0.827789946 |
| M05 - M07 | -0.379587292 | 0.704251795 | 0.831038427 |
| M01 - M20 | -0.36895953 | 0.71215789 | 0.836571015 |
| M20 - M27 | 0.367339866 | 0.713365521 | 0.836729483 |
| M25 - M39 | -0.369945248 | 0.711423286 | 0.836968572 |
| M07 - M10 | -0.370891301 | 0.710718494 | 0.837402456 |
| M29 - M32 | -0.362120355 | 0.717262093 | 0.840036686 |
| M31 - M40 | -0.353688131 | 0.723572618 | 0.841112731 |
| M15 - M32 | 0.352206326 | 0.724683533 | 0.84115053 |
| M28 - M37 | -0.355641867 | 0.722108786 | 0.841920557 |
| M24 - M38 | 0.357054254 | 0.72105119 | 0.841946001 |
| M21 - M28 | 0.353890166 | 0.723421197 | 0.842191841 |
| M10 - M12 | -0.357557176 | 0.720674731 | 0.842768051 |
| M27 - M35 | 0.344245161 | 0.730661922 | 0.846829567 |
| M01 - M35 | 0.34228365 | 0.732137438 | 0.847280715 |
| M20 - M39 | -0.340453438 | 0.733515081 | 0.847617427 |
| M09 - M17 | 0.328072434 | 0.742856896 | 0.854614128 |
| M25 - M31 | -0.328511708 | 0.742524793 | 0.855493853 |
| M02 - M38 | 0.328697124 | 0.742384629 | 0.856597648 |
| M03 - M34 | -0.319658988 | 0.749226852 | 0.860672967 |
| M27 - M36 | 0.317660315 | 0.750742617 | 0.861145943 |
| M01 - M36 | 0.315961597 | 0.752031657 | 0.861357846 |
| M30 - M35 | 0.306193672 | 0.75945719 | 0.868587402 |
| M12 - M17 | -0.301848568 | 0.762767504 | 0.871096125 |
| M20 - M31 | -0.298826333 | 0.765072557 | 0.872451162 |
| M05 - M11 | 0.288603517 | 0.772884804 | 0.878790301 |
| M07 - M33 | -0.289782741 | 0.771982452 | 0.879045712 |
| M06 - M32 | 0.27679456 | 0.781937859 | 0.88521267 |
| M07 - M16 | 0.278234584 | 0.780832289 | 0.885245909 |
| M30 - M36 | 0.279664956 | 0.779734567 | 0.885288154 |
| M02 - M31 | 0.273238538 | 0.784669858 | 0.8870181 |
| M15 - M33 | 0.27015431 | 0.787041541 | 0.88841158 |
| M26 - M40 | -0.266836041 | 0.789595403 | 0.89000638 |
| M39 - M40 | -0.264678322 | 0.791257278 | 0.890592608 |
| M18 - M38 | 0.260573636 | 0.794421323 | 0.891580765 |
| M02 - M26 | 0.261856194 | 0.793432314 | 0.891753897 |
| M24 - M31 | 0.251215104 | 0.801647808 | 0.898398405 |
| M03 - M14 | 0.245026563 | 0.806435864 | 0.902467681 |
| M22 - M27 | -0.241581389 | 0.809104545 | 0.90286344 |
| M01 - M22 | 0.240062009 | 0.810282186 | 0.902885864 |
| M19 - M38 | 0.242301781 | 0.808546334 | 0.903533152 |
| M02 - M39 | 0.235172804 | 0.814074613 | 0.905817687 |
| M07 - M29 | 0.22798582 | 0.819657264 | 0.909434803 |
| M10 - M15 | -0.225825585 | 0.821337077 | 0.910004148 |
| M03 - M09 | 0.228349813 | 0.819374302 | 0.910415891 |
| M06 - M12 | -0.211649661 | 0.832380363 | 0.91962703 |
| M28 - M35 | -0.213061017 | 0.831279371 | 0.919713346 |
| M10 - M32 | 0.209819706 | 0.833808392 | 0.919901762 |
| M21 - M35 | 0.203846191 | 0.838473695 | 0.923742206 |
| M22 - M30 | -0.201797434 | 0.840075088 | 0.924201084 |
| M21 - M36 | 0.199798444 | 0.841638218 | 0.924616634 |
| M24 - M26 | 0.193720925 | 0.846394407 | 0.928533948 |
| M20 - M26 | -0.166690675 | 0.867613443 | 0.929585832 |
| M27 - M37 | 0.160166901 | 0.872749602 | 0.929979084 |
| M01 - M37 | 0.158404058 | 0.874138416 | 0.930188219 |
| M02 - M19 | 0.190162386 | 0.849181885 | 0.930283526 |
| M19 - M40 | -0.173430019 | 0.862313431 | 0.930296647 |
| M05 - M29 | -0.161054259 | 0.872050666 | 0.930505498 |
| M02 - M18 | 0.167109331 | 0.867284024 | 0.930511057 |
| M25 - M38 | -0.188372393 | 0.850584739 | 0.93051346 |
| M18 - M31 | 0.168164528 | 0.866453846 | 0.930900826 |
| M04 - M06 | 0.169165185 | 0.865666713 | 0.931337981 |
| M24 - M39 | 0.161434104 | 0.871751508 | 0.931460515 |
| M25 - M26 | -0.173522062 | 0.862241088 | 0.931506993 |
| M02 - M24 | 0.162709771 | 0.870746953 | 0.931663407 |
| M06 - M08 | -0.185464978 | 0.852864347 | 0.931700547 |
| M18 - M26 | 0.154583362 | 0.877149788 | 0.932121028 |
| M28 - M36 | -0.153014894 | 0.87838653 | 0.932165297 |
| M36 - M37 | -0.169484787 | 0.865415338 | 0.932353541 |
| M22 - M28 | 0.180037327 | 0.857123264 | 0.93243535 |
| M06 - M33 | 0.181439757 | 0.856022415 | 0.932538385 |
| M20 - M38 | -0.173628014 | 0.862157814 | 0.93270887 |
| M02 - M23 | -0.182578631 | 0.855128653 | 0.932867621 |
| M35 - M37 | -0.174547601 | 0.861435124 | 0.933221385 |
| M38 - M39 | -0.174967981 | 0.861104793 | 0.934160972 |
| M07 - M32 | -0.175193041 | 0.860927952 | 0.93526992 |
| M19 - M31 | 0.142961821 | 0.886320334 | 0.938032375 |
| M21 - M22 | 0.143554257 | 0.885852464 | 0.938811035 |
| M09 - M13 | -0.137542691 | 0.890601851 | 0.941286509 |
| M06 - M10 | 0.129459719 | 0.896993896 | 0.941662502 |
| M18 - M40 | -0.127750202 | 0.89834666 | 0.941815047 |
| M19 - M26 | 0.13433721 | 0.89313593 | 0.94268745 |
| M13 - M14 | 0.129460833 | 0.896993015 | 0.942930662 |
| M24 - M40 | -0.129643901 | 0.896848168 | 0.944050704 |
| M05 - M16 | -0.123069764 | 0.902051854 | 0.944430129 |
| M31 - M38 | 0.120764413 | 0.903877638 | 0.945073134 |
| M32 - M33 | -0.129904959 | 0.896641621 | 0.945108736 |
| M04 - M12 | -0.118528503 | 0.905648912 | 0.945657499 |
| M30 - M37 | 0.10963926 | 0.912695473 | 0.951741269 |
| M06 - M15 | -0.098841466 | 0.921264145 | 0.95176958 |
| M02 - M40 | 0.099866927 | 0.920449974 | 0.952189628 |
| M03 - M13 | 0.100750705 | 0.91974836 | 0.952727385 |
| M22 - M37 | -0.106626153 | 0.91508557 | 0.952959605 |
| M12 - M15 | 0.102979322 | 0.917979383 | 0.953427321 |
| M18 - M39 | 0.104077516 | 0.917107833 | 0.953792147 |
| M08 - M12 | -0.100841165 | 0.919676548 | 0.953919824 |
| M26 - M39 | -0.081679914 | 0.934901252 | 0.962035589 |
| M01 - M21 | 0.082806199 | 0.93400564 | 0.962383618 |
| M21 - M27 | -0.084325579 | 0.932797576 | 0.962410197 |
| M19 - M39 | 0.075476357 | 0.939835708 | 0.965839068 |
| M10 - M33 | 0.073510876 | 0.94139959 | 0.966173263 |
| M19 - M24 | -0.068759281 | 0.945181231 | 0.968779711 |
| M31 - M39 | -0.066284227 | 0.947151541 | 0.969525199 |
| M27 - M30 | 0.059928205 | 0.952212816 | 0.973428567 |
| M01 - M30 | 0.058112199 | 0.953659258 | 0.973631179 |
| M21 - M37 | 0.055324891 | 0.955879633 | 0.974622371 |
| M04 - M08 | -0.035329506 | 0.971816995 | 0.980617408 |
| M08 - M15 | 0.035958865 | 0.971315159 | 0.981380601 |
| M26 - M38 | 0.042041132 | 0.966465908 | 0.981566938 |
| M18 - M19 | 0.032018323 | 0.974457438 | 0.982011372 |
| M16 - M29 | -0.042467957 | 0.966125656 | 0.982500667 |
| M21 - M30 | -0.036035256 | 0.971254247 | 0.982591845 |
| M26 - M31 | -0.039077905 | 0.968828277 | 0.982686679 |
| M18 - M24 | -0.027353358 | 0.978177899 | 0.983220053 |
| M09 - M14 | -0.036297617 | 0.97104505 | 0.983656024 |
| M22 - M36 | 0.042542634 | 0.966066125 | 0.983722686 |
| M22 - M35 | 0.028028851 | 0.97763914 | 0.983946489 |
| M35 - M36 | 0.022557375 | 0.982003345 | 0.985794863 |
| M04 - M15 | 0.019997779 | 0.984045144 | 0.986574823 |
| M20 - M25 | -0.000574272 | 0.999541798 | 0.999541798 |
| M01 - M27 | -0.001698718 | 0.99864462 | 0.999926577 |

Appendix S2 **Summary of additional linear model**

Instead of number of fathers we used the effective mating frequency (me) as predictor variable. As seen from the model output none of the variables had a significant influence on mean SVL per matriline.

Call:

lm(formula = av_size ~ n + me + av_days, data = g_new_all)

Residuals:

Min 1Q Median 3Q Max

-0.88133 -0.23692 0.01119 0.27032 0.86698

Coefficients:

Estimate Std. Error t value Pr(>|t|)

(Intercept) 13.444997 0.877656 15.319 <2e-16 ***

n 0.020688 0.012594 1.643 0.109

me -0.168052 0.116968 -1.437 0.159

av_days 0.005687 0.007940 0.716 0.478

---

Signif. codes: 0 ‘***’ 0.001 ‘**’ 0.01 ‘*’ 0.05 ‘.’ 0.1 ‘ ’ 1

Residual standard error: 0.4399 on 36 degrees of freedom

Multiple R-squared: 0.09253, Adjusted R-squared: 0.01691

F-statistic: 1.224 on 3 and 36 DF, p-value: 0.3152

Appendix S3 **Summary of GAM**

The response variable, mean BCI, was modelled with a generalized additive model. The mean developmental team was used without smoother as it showed a linear relationship with BCI. The number off offspring and number of fathers were used with a cubic regression spline (cs). The number of fathers ranged from 1 to 5, therefore, we reduced number of knots to 3.

Family: gaussian

Link function: identity

Formula:

av_bci ~ av_days + s(n, bs = "cs") + s(n_fathers, bs = "cs",

k = 3)

Parametric coefficients:

Estimate Std. Error t value Pr(>|t|)

(Intercept) 0.1463089 0.0295109 4.958 1.51e-05 ***

av_days 0.0004605 0.0002689 1.712 0.095 .

---

Signif. codes: 0 ‘***’ 0.001 ‘**’ 0.01 ‘*’ 0.05 ‘.’ 0.1 ‘ ’ 1

Approximate significance of smooth terms:

edf Ref.df F p-value

s(n) 5.433e-05 9 0 0.435

s(n_fathers) 4.678e-05 2 0 0.643

R-sq.(adj) = 0.0472 Deviance explained = 7.16%

-REML = -98.348 Scale est. = 0.00024243 n = 40


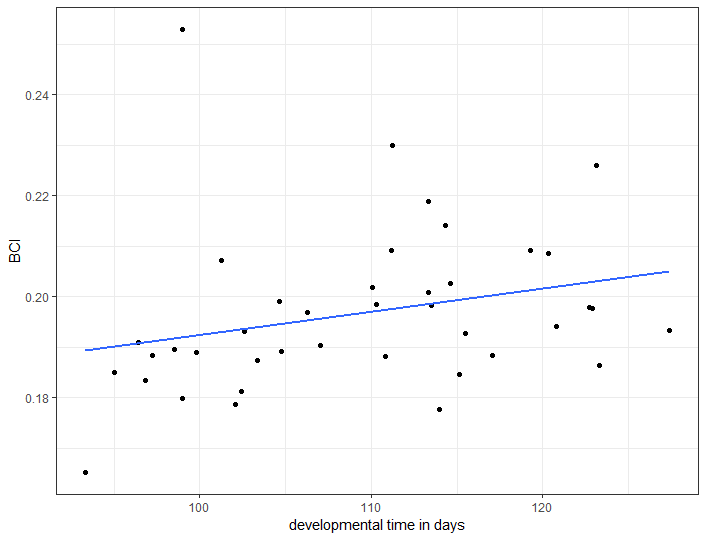


**Figure S5** Results of the GAM. The developmental time showed a positive linear trend on the BCI, but was not significant.
